# Multilevel quadrature for elliptic problems on random domains by the coupling of FEM and BEM

**DOI:** 10.1007/s40072-021-00214-w

**Published:** 2021-10-13

**Authors:** Helmut Harbrecht, Marc Schmidlin

**Affiliations:** Departement Mathematik und Informatik, Universität Basel, Spiegelgasse 1, 4051 Basel, Switzerland

**Keywords:** Uncertainty quantification, Random domain, Regularity, Multilevel method, FEM-BEM coupling, 35R60, 65N30, 65N38

## Abstract

Elliptic boundary value problems which are posed on a random domain can be mapped to a fixed, nominal domain. The randomness is thus transferred to the diffusion matrix and the loading. While this domain mapping method is quite efficient for theory and practice, since only a single domain discretisation is needed, it also requires the knowledge of the domain mapping. However, in certain applications, the random domain is only described by its random boundary, while the quantity of interest is defined on a fixed, deterministic subdomain. In this setting, it thus becomes necessary to compute a random domain mapping on the whole domain, such that the domain mapping is the identity on the fixed subdomain and maps the boundary of the chosen fixed, nominal domain on to the random boundary. To overcome the necessity of computing such a mapping, we therefore couple the finite element method on the fixed subdomain with the boundary element method on the random boundary. We verify on one hand the regularity of the solution with respect to the random domain mapping required for many multilevel quadrature methods, such as the multilevel quasi-Monte Carlo quadrature using Halton points, the multilevel sparse anisotropic Gauss–Legendre and Clenshaw–Curtis quadratures and multilevel interlaced polynomial lattice rules. On the other hand, we derive the coupling formulation and show by numerical results that the approach is feasible.

## Introduction

Many practical problems in science and engineering lead to elliptic boundary value problems for an unknown function. Their numerical treatment by e.g. finite difference or finite element methods is in general well understood provided that the input parameters are given exactly. This, however, is often not the case in practical applications.

If a statistical description of the input data is available, one can mathematically describe data and solutions as random fields and aim at the computation of corresponding deterministic statistics of the unknown random solution. The present article is dedicated to the treatment of uncertainties in the description of the computational domain. Applications are, besides traditional engineering, for example uncertain domains which are derived from inverse methods such as tomography. In recent years, this situation has become of growing interest: In [44] the so-called domain mapping method was introduced as an approach to describe and solve boundary value problems on random domains; this was extended in [42], where the same authors used the domain mapping method to consider an advection-diffusion equation on a random tube shaped domain. Recently, the domain mapping method has also been considered for partial differential equations on random bulk and surface domains in [6]. The domain mapping method was rigorously analysed for elliptic partial differential equations on random domains in [5, 28] and for acoustic scattering problems in [35], where analytic dependency of the solution on the random domain mapping with regard to the energy norm has been verified. Moreover, the use of Multilevel Monte Carlo quadrature together with the domain mapping approach has been considered in [40].

Apart from the domain mapping method, other methods of describing and solving boundary value problems on random domains have also been considered; by the necessity of being domain mapping free, they can only be directly posed and solved on realisations of the random domain or its boundary. In [4] for example, a fictious domain formulation is used, enabling the prescription of boundary data for the Poisson problem at a random boundary inside the domain of computation yielding a random domain. An approach based on describing a random domain as a random mesh with deterministic connectivity was considered in [39]. While in [38] a random domain is described by randomly perturbing the boundary, which suffices since a surface integral equation formulation is used. More recently, a kind of boundary mapping method based on Jordan curves for a boundary integral equation formulation of the Laplacian on simply connected random domains in ${\mathbb {R}}^2$ was considered in [33], where it is shown that the solution of the boundary integral equation depends analytically on the boundary mapping.

A further alternative approach based on shape calculus is considered in [29, 31] for elliptic boundary value problems. Describing the random domain by a random perturbation of a fixed domains boundary one arrives at a shape Taylor expansion, with which approximations of the expectation and correlation of the solution are computed requiring the solving of tensor produt boundary value problems.

In this article, we are going to focus on the domain mapping method. Given enough spatial regularity of the random domain mapping, we first prove that the solution is analytically dependent on the random domain mapping also in the $H^{\tau +1}(D)$-norm for $\tau \in {\mathbb {N}}$. The key idea of the domain mapping method is to map the boundary value problem

1 $$\begin{aligned} -\Delta _{\mathbf {x}}u[\omega ] = f \text { in }{\mathfrak {D}}[\omega ], \quad u[\omega ] = 0 \text { on }\partial {\mathfrak {D}}[\omega ], \end{aligned}$$

which is posed on a random domain

$$\begin{aligned} {\mathfrak {D}}[\omega ] \mathrel {\mathrel {\mathop :}=}{\mathbf {V}}[\omega ](D) \subset {\mathbb {R}}^d \end{aligned}$$

defined by the *random domain mapping* ${\mathbf {V}}[\omega ] :D \rightarrow {\mathbb {R}}^d$, on a fixed, nominal *reference* domain $D \subset {\mathbb {R}}^d$, back onto that fixed reference domain *D*. Thus, the randomness is transferred to the diffusion matrix and the loading of the boundary value problem

2 $$\begin{aligned} -{{\,\mathrm{div}\,}}_{\mathbf {x}}\big ({\hat{{\mathbf {A}}}}[\omega ] \nabla _{\mathbf {x}}{\hat{u}}[\omega ]\big ) = {\hat{f}}[\omega ] \text { in }D, \quad {\hat{u}}[\omega ] = 0 \text { on }\partial D. \end{aligned}$$

Herein, it holds

3 $$\begin{aligned} {\hat{{\mathbf {A}}}}[\omega ] \mathrel {\mathrel {\mathop :}=}\big ({\mathbf {J}}[\omega ]^{\mathsf {T}}{\mathbf {J}}[\omega ]\big )^{-1} \det {\mathbf {J}}[\omega ] \quad \text {and}\quad {\hat{f}}[\omega ] \mathrel {\mathrel {\mathop :}=}\big (f \circ {\mathbf {V}}[\omega ]\big ) \det {\mathbf {J}}[\omega ], \end{aligned}$$

where ${\mathbf {J}}[\omega ]$ denotes the Jacobian of the field ${\mathbf {V}}[\omega ] :D \rightarrow {\mathfrak {D}}[\omega ]$

4 $$\begin{aligned} {\mathbf {J}}[\omega ]({\mathbf {x}}) \mathrel {\mathrel {\mathop :}=}{{\,\mathrm{D}\,}}_{\mathbf {x}}{\mathbf {V}}[\omega ]({\mathbf {x}}) . \end{aligned}$$

and ${\hat{u}}[\omega ]$ is connected to $u[\omega ]$ by ${\hat{u}}[\omega ] \mathrel {\mathrel {\mathop :}=}u[\omega ] \circ {\mathbf {V}}[\omega ]$.^1^ As one arrives at a formulation of a boundary value problem with random data for the diffusion matrix and the loading, the result that the solution in the $H^{\tau +1}(D)$-norm is analytically dependent on the random data for the diffusion matrix and the loading follows essentially from [30]. Therefore, we have to verify that the diffusion matrix and the loading depend analytically on the domain mapping with respect to appropriate norms. This analytical dependence is then sufficient to justify using multilevel versions of many quadrature methods to evaluate quantity of interest expressions of the form

$$\begin{aligned} {{\,\mathrm{QoI}\,}}(u) = \int _\varOmega {\mathcal {F}}\big ({\hat{u}}[\omega ]\big ) {{\,\mathrm{d}\,}}\!{\mathbb {P}}[\omega ] , \end{aligned}$$

where ${\mathcal {F}}:H^{\sigma }(D) \rightarrow {\mathcal {X}}$ is a smooth, possibly non-linear, operator into some Banach space ${\mathcal {X}}$, with $\sigma \le 1$,^2^ and the integral is over the probability space with sample space $\varOmega$ and measure ${\mathbb {P}}$, cf. [27].

Indeed, when the random domain mapping is given in a parametric form

$$\begin{aligned} {\mathbf {V}}:\square \rightarrow (D \rightarrow {\mathbb {R}}^d),\quad {\mathbf {y}}\mapsto {\mathbf {V}}[{\mathbf {y}}] , \end{aligned}$$

where ${\mathbf {y}}\in \square = \left[ {{-\frac{1}{2}}, \frac{1}{2}}\right] ^{{\mathbb {N}}^*}$ is a sequence of independent and identically uniformly distributed random variables with its pushforward measure denoted by ${\mathbb {P}}_{\mathbf {y}}$, the quantity of interest expression may be written as an infinite-dimensional integral,

$$\begin{aligned} {{\,\mathrm{QoI}\,}}(u) = \int _\square {\mathcal {F}}\big ({\hat{u}}[{\mathbf {y}}]\big ) {{\,\mathrm{d}\,}}\!{\mathbb {P}}_{\mathbf {y}}. \end{aligned}$$

Then, bounds on the partial derivative of ${\hat{u}}$ of the form

$$\begin{aligned} \big \Vert \partial _{\mathbf {y}}^{\varvec{\alpha }}{\hat{u}}[{\mathbf {y}}]\big \Vert _{H^\sigma (D)} \le \big |{\varvec{\alpha }}\big |! c^{|{{\varvec{\alpha }}}|+1} {\varvec{\gamma }}^{\varvec{\alpha }}, \end{aligned}$$

where ${\varvec{\gamma }}\in \ell ^1({{\mathbb {N}}})$ is a sequence relating to the decay of the importance of the sequence of parameters ${\mathbf {y}}$ with respect to the domain mapping ${\mathbf {V}}$, imply similar estimates for the integrand ${\mathcal {F}}\big (u[{\mathbf {y}}]\big )$.

Given that these estimates hold for all finitely supported multi-indices with a sufficiently fast decaying ${\varvec{\gamma }}$, this justifies approximating a truncation of the infinite-dimensional integral with the quasi-Monte Carlo quadrature with Halton points, see e.g. [22, 28, 43] and the sparse anisotropic Gauss–Legendre and Clenshaw–Curtis quadratures, see e.g. [18, 21], yielding provable error rates. Similarly, if these estimates only hold for all finitely supported multi-indices ${\varvec{\alpha }}\in \{0, 1, \ldots , s\}^{{\mathbb {N}}^*}$ for some $s \ge 1$ with a sufficiently fast decaying ${\varvec{\gamma }}$, one may instead consider higher-order quasi-Monte Carlo quadratures, such as interlaced polynomial lattice rules, see e.g. [10, 11]. In general, these types of bounds on the partial derivative of ${\hat{u}}$ will require an analytic dependence of the solution in the $H^{\sigma }(D)$-norm on the domain mapping, as they all include bounds for *mixed* derivatives of ${\hat{u}}$ in the integration variables.

When one wants to consider the multilevel versions of the previously mentioned quadratures, one is considering a sparse grid combination technique of the quadrature methods and the spatial discretisation. This requires *mixed* smoothness between the smoothness in the integration variables and the spatial smoothness, see e.g. [18, 19, 27], which means bounds of the form

$$\begin{aligned} \big \Vert \partial _{\mathbf {y}}^{\varvec{\alpha }}{\hat{u}}[{\mathbf {y}}]\big \Vert _{H^{\tau + 1}(D)} \le \big |{\varvec{\alpha }}\big |! c^{|{{\varvec{\alpha }}}|+1} {\varvec{\gamma }}^{\varvec{\alpha }}. \end{aligned}$$

This type of mixed smoothness follows, when the solution in the $H^{\tau + 1}(D)$-norm is analytically dependent on the domain mapping.

However, while the random domain mapping approach is mathematically natural, it is not necessarily the setting that is directly encountered in practical applications. This mainly stems from the fact that the random domain mapping does not only describe the random domains themselves but also includes a specific point correspondence between the domain realisations. In applications often only a description of the random boundary might be known, however in such cases the quantity of interest

5 $$\begin{aligned} {{\,\mathrm{QoI}\,}}(u) = \int _\varOmega {\mathcal {F}}\big (u[\omega ]\vert _B\big ) {{\,\mathrm{d}\,}}\!{\mathbb {P}}[\omega ] \end{aligned}$$

is generally sought on a *deterministic* subdomain, *B*, which almost surely is a subset of the domain realisations, where ${\mathcal {F}}:H^{\sigma }(B) \rightarrow {\mathcal {X}}$ is a smooth, possibly non-linear, operator into some Banach space ${\mathcal {X}}$.^3^ Therefore, it is then necessary to be able to transform the description of the random domains given by a description of the random boundary and the specification of the subdomain into the form of a random domain mapping. To be able to justify the use of the multilevel versions of the above mentioned quadrature methods, we therefore require that the method for transforming the description of the random boundary into a random domain mapping is an analytic map from boundary descriptions to domain mappings. This then implies that the solution in the $H^{\tau + 1}(D)$-norm is also analytically dependent on the description of the random boundary. In [44], the authors consider using the vector-valued Laplace equation to compute such a random domain mapping. If more structure is given, for example when the random domains are described by star-shaped boundaries or more generally when they are directly given by a boundary mapping from a nominal boundary, one may also consider other approaches, such as transfinite interpolation techniques, see e.g. [14–16], to extend the mapping onto the whole reference domain.

To overcome the necessity of computing such a random domain mapping in this setting, we propose to compute the quantity of interest by performing the calculations on the realisations of the random domains. However, in our setting, care then must be taken that the discretisation chosen is regular enough to ensure that the spatially discretised problems inherit sufficient regularity with respect to their dependence on the boundary description, such that the multilevel quadrature method stays viable. Therefore, we choose to sidestep the generation of a mesh on the random part of the domain ${\mathfrak {D}}[\omega ] \setminus B$ completely by coupling the finite element method with the boundary element method for the spatial approximation as follows: we apply finite elements on the subdomain *B* and treat the rest of the domain by a boundary element method. This is also advantageous, since large domain deformations on coarse discretisations can be handled more easily, as we do not need to mesh the random part of the domain but only its boundary. Moreover, such an approach may also be useful when computing on an unbounded domain.

The contribution of this article is thus twofold:

- First, we extend results regarding the regularity of the domain mapping method for elliptic partial differential equations on random domains from [5, 28] to allow for higher spatial smoothness, which justifies many multilevel quadrature methods.
- Second, we propose and discuss using a coupling of the finite element method and the boundary element method as the spatial discretisation in a multilevel quadrature method. This yields an efficient method that only requires a domain mapping to exist, but does neither need to know it nor need to compute it. Thus, it is very applicable to practical problems, where only knowledge of a random boundary description is available, for example, from nondestructive measurements.

The rest of this article is organised as follows. Section 2 is dedicated to the mathematical formulation of the problem under consideration. The problem’s regularity is studied in Sect. 3. Here, we provide estimates in stronger spatial norms which are needed for many multilevel accelerated quadrature methods. The coupling of finite elements and boundary elements is the topic of Sect. 4. The multilevel quadrature method for the approximation of quantities of interest of the solution of the boundary value problem on random domains is then discussed in Sect. 5. Numerical experiments are carried out in Sect. 6. Finally, we state concluding remarks in Sect. 7.

## Notation and model problem

Before we complete the mathematical setting of our model problem, we will introduce the notations used throughout the rest of the article. Especially, for the regularity considerations in Sect. 3 some of the notation—and the choice of a certain weighting in the Sobolev–Bochner norms—helps keep formulas somewhat more concise and compact.

### Notation and precursory remarks

We use ${\mathbb {N}}$ to denote the natural numbers including 0 and ${\mathbb {N}}^*$ when excluding 0. For a sequence of natural numbers, ${\varvec{\alpha }}= \big \{\alpha _n\big \}_{n \in {\mathbb {N}}^*} \in {\mathbb {N}}^{{\mathbb {N}}^*}$, we define the support of the sequence as

$$\begin{aligned} {{\,\mathrm{supp}\,}}{\varvec{\alpha }}= \big \{n \in {\mathbb {N}}^* \,|\, \alpha _n \ne 0\big \} \end{aligned}$$

and say that ${\varvec{\alpha }}$ is finitely supported, if ${{\,\mathrm{supp}\,}}{\varvec{\alpha }}$ is of finite cardinality. Then, ${\mathbb {N}}^{{\mathbb {N}}^*}_f$ denotes the set of finitely supported sequences of natural numbers and we refer to its elements as multi–indices. Furthermore, for all $m \in {\mathbb {N}}^*$ we will identify the elements ${\varvec{\alpha }}= \big (\alpha _1, \ldots , \alpha _m\big ) \in {\mathbb {N}}^m$ with their extension by zero into ${\mathbb {N}}^{{\mathbb {N}}^*}_f$, that is ${\varvec{\alpha }}= \big (\alpha _1, \ldots , \alpha _m, 0, \ldots \big )$. Thus, by this identification, all notations defined for elements of ${\mathbb {N}}^{{\mathbb {N}}^*}_f$ also carry over to the elements of ${\mathbb {N}}^m$ and we also refer to elements of ${\mathbb {N}}^m$ as multi–indices.

For multi-indices ${\varvec{\alpha }}= \big \{\alpha _n\big \}_{n \in {\mathbb {N}}^*}, {\varvec{\beta }}= \big \{\beta _n\big \}_{n \in {\mathbb {N}}^*} \in {\mathbb {N}}^{{\mathbb {N}}^*}_f$ and a sequence of real numbers ${\varvec{\gamma }}= \big \{\gamma _n\big \}_{n \in {\mathbb {N}}^*} \in {\mathbb {R}}^{{\mathbb {N}}^*}$, we use the following common notations:

$$\begin{aligned} \big |{\varvec{\alpha }}\big |&\mathrel {\mathrel {\mathop :}=}\sum _{n \in {{\,\mathrm{supp}\,}}{\varvec{\alpha }}} \alpha _n ,&{\varvec{\alpha }}!&\mathrel {\mathrel {\mathop :}=}\prod _{n \in {{\,\mathrm{supp}\,}}{\varvec{\alpha }}} \alpha _n! , \\ \left( {\begin{array}{c}{\varvec{\alpha }}\\ {\varvec{\beta }}\end{array}}\right)&\mathrel {\mathrel {\mathop :}=}\prod _{n \in {{\,\mathrm{supp}\,}}{\varvec{\alpha }}\cup {{\,\mathrm{supp}\,}}{\varvec{\beta }}} \left( {\begin{array}{c}\alpha _n\\ \beta _n\end{array}}\right) ,&{\varvec{\gamma }}^{\varvec{\alpha }}&\mathrel {\mathrel {\mathop :}=}\prod _{n \in {{\,\mathrm{supp}\,}}{\varvec{\alpha }}} \gamma _n^{\alpha _n} . \end{aligned}$$

Furthermore, we say that ${\varvec{\alpha }}\le {\varvec{\beta }}$ holds, when $\alpha _j \le \beta _j$ holds for all $j \in {{\,\mathrm{supp}\,}}{\varvec{\alpha }}\cup {{\,\mathrm{supp}\,}}{\varvec{\beta }}$, and ${\varvec{\alpha }}< {\varvec{\beta }}$, when ${\varvec{\alpha }}\le {\varvec{\beta }}$ and ${\varvec{\alpha }}\ne {\varvec{\beta }}$ hold.

Subsequently, we will always equip ${\mathbb {R}}^m$ with the norm $\big \Vert \cdot \big \Vert _2$ induced by the canonical inner product $\langle \cdot ,\cdot \rangle$ and ${\mathbb {R}}^{m \times m}$ with the induced norm $\big \Vert \cdot \big \Vert _2$. Moreover, when considering ${\mathbb {R}}^m$ itself or an open domain ${\mathcal {D}}\subset {\mathbb {R}}^m$ as a measure space we always equip it with the Lebesgue measure. Similarly, we always equip ${\mathbb {N}}$ and ${\mathbb {N}}^*$ with the counting measure, when considering them as measure spaces.

Let ${\mathcal {X}}$, ${\mathcal {X}}_1, \ldots , {\mathcal {X}}_r$ and ${\mathcal {Y}}$ be Banach spaces, then we denote the Banach space of bounded, linear maps from ${\mathcal {X}}$ to ${\mathcal {Y}}$ as ${\mathcal {B}}({\mathcal {X}}; {\mathcal {Y}})$; furthermore, we recursively define

$$\begin{aligned} {\mathcal {B}}^0({\mathcal {X}}; {\mathcal {Y}}) \mathrel {\mathrel {\mathop :}=}{\mathcal {Y}}\quad \text {and}\quad {\mathcal {B}}^{r+1}({\mathcal {X}}; {\mathcal {Y}}) \mathrel {\mathrel {\mathop :}=}{\mathcal {B}}\big ({\mathcal {X}}; {\mathcal {B}}^r({\mathcal {X}}; {\mathcal {Y}})\big ) . \end{aligned}$$

For ${\mathbf {T}}\in {\mathcal {B}}^r({\mathcal {X}}; {\mathcal {Y}})$ and ${\mathbf {v}}_j \in {\mathcal {X}}$ we use the shorthand notation

$$\begin{aligned} {\mathbf {T}}{\mathbf {v}}_1 \cdots {\mathbf {v}}_r \mathrel {\mathrel {\mathop :}=}{\mathbf {T}}({\mathbf {v}}_1, \ldots , {\mathbf {v}}_r) \in {\mathcal {Y}}. \end{aligned}$$

For a given Banach space ${\mathcal {X}}$ and a complete measure space ${\mathcal {M}}$ with measure $\mu$ the space $L_\mu ^p({\mathcal {M}}; {\mathcal {X}})$ for $1 \le p \le \infty$ denotes the Bochner space, see [34], which contains all equivalence classes of strongly measurable functions $v :{\mathcal {M}}\rightarrow {\mathcal {X}}$ with finite norm

$$\begin{aligned} \big \Vert v\big \Vert _{p, {\mathcal {M}}; {\mathcal {X}}} \mathrel {\mathrel {\mathop :}=}\big \Vert v\big \Vert _{L_\mu ^p({\mathcal {M}}; {\mathcal {X}})} \mathrel {\mathrel {\mathop :}=}{\left\{ \begin{array}{ll} \bigg [\displaystyle \int _{\mathcal {M}}\big \Vert v(x)\big \Vert _{{\mathcal {X}}}^p {{\,\mathrm{d}\,}}\,\mu (x)\bigg ]^{1/p} , &{} p < \infty , \\ \displaystyle \mathop {\hbox {ess sup}}\limits _{x \in {\mathcal {M}}} \big \Vert v(x)\big \Vert _{{\mathcal {X}}} , &{} p = \infty . \end{array}\right. } \end{aligned}$$

A function $v :{\mathcal {M}}\rightarrow {\mathcal {X}}$ is strongly measurable if there exists a sequence of countably–valued measurable functions $v_n :{\mathcal {M}}\rightarrow {\mathcal {X}}$, such that for almost every $m \in {\mathcal {M}}$ we have $\lim _{n \rightarrow \infty } v_n(m) = v(m)$. Note that, for finite measures $\mu$, we also have the usual inclusion $L_\mu ^p({\mathcal {M}}; {\mathcal {X}}) \supset L_\mu ^q({\mathcal {M}}; {\mathcal {X}})$ for $1 \le p < q \le \infty$.

For a given Banach space ${\mathcal {X}}$ and an open domain ${\mathcal {D}}\subset {\mathbb {R}}^d$, with $d \in {\mathbb {N}}^*$, the space $W^{\eta , p}({\mathcal {D}}; {\mathcal {X}})$ for $\eta \in {\mathbb {N}}$ and $1 \le p \le \infty$ denotes the Sobolev–Bochner space, which contains all equivalence classes of strongly measurable functions $v :{\mathcal {D}}\rightarrow {\mathcal {X}}$, such that the function itself and all weak derivatives up to total order $\eta$ are in $L^p({\mathcal {D}}; {\mathcal {X}})$ with the norm

$$\begin{aligned} \big \Vert v\big \Vert _{\eta , p, {\mathcal {D}}; {\mathcal {X}}} \mathrel {\mathrel {\mathop :}=}\big \Vert v\big \Vert _{W^{\eta , p}({\mathcal {D}}; {\mathcal {X}})} \mathrel {\mathrel {\mathop :}=}\sum _{\left| {{\varvec{\alpha }}}\right| \le \eta } \frac{1}{{\varvec{\alpha }}!} \big \Vert \partial _{\mathbf {x}}^{\varvec{\alpha }}v\big \Vert _{p, {\mathcal {D}}; {\mathcal {X}}} . \end{aligned}$$

Moreover, $W_0^{\eta , p}({\mathcal {D}}; {\mathcal {X}})$ denotes the closure of the linear subspace of smooth functions with compact support, $C_c^\infty ({\mathcal {D}}; {\mathcal {X}})$, in $W^{\eta , p}({\mathcal {D}}; {\mathcal {X}})$ and we also define $H^\eta ({\mathcal {D}}; {\mathcal {X}}) \mathrel {\mathrel {\mathop :}=}W^{\eta , 2}({\mathcal {D}}; {\mathcal {X}})$ and $H_0^\eta ({\mathcal {D}}; {\mathcal {X}}) \mathrel {\mathrel {\mathop :}=}W_0^{\eta , 2}({\mathcal {D}}; {\mathcal {X}})$. As usual, we use $C^{\omega }({\mathcal {D}}; {\mathcal {X}})$ to denote the real analytic functions^4^ from ${\mathcal {D}}$ to ${\mathcal {X}}$ and $C^{k, s}({\mathcal {D}}; {\mathcal {X}})$ to denote the Hölder spaces. For a bi-Lipschitz function $v :{\mathcal {D}}\rightarrow {\mathcal {X}}$ we denote its bi-Lipschitz constants by

$$\begin{aligned} \big |v\big |_{{\underline{Lip}}({\mathcal {D}}; {\mathcal {X}})}&\mathrel {\mathrel {\mathop :}=}\mathop {\hbox {ess inf}}\limits _{{\mathbf {x}}, {\mathbf {z}}\in {\mathcal {D}},\, {\mathbf {x}}\ne {\mathbf {z}}} \frac{\big \Vert v({\mathbf {x}}) - v({\mathbf {z}})\big \Vert _{{\mathcal {X}}}}{\big \Vert {\mathbf {x}}- {\mathbf {y}}\big \Vert }, \\ \big |v\big |_{{\overline{Lip}}({\mathcal {D}}; {\mathcal {X}})}&\mathrel {\mathrel {\mathop :}=}\mathop {\hbox {ess sup}}\limits _{{\mathbf {x}}, {\mathbf {z}}\in {\mathcal {D}},\, {\mathbf {x}}\ne {\mathbf {z}}} \frac{\big \Vert v({\mathbf {x}}) - v({\mathbf {z}})\big \Vert _{{\mathcal {X}}}}{\big \Vert {\mathbf {x}}- {\mathbf {y}}\big \Vert } . \end{aligned}$$

In the notation for the Bochner, Sobolev–Bochner and Hölder spaces, we may omit specifying the Banach space ${\mathcal {X}}$ when ${\mathcal {X}}= {\mathbb {R}}$. Especially, $H^{-\eta }({\mathcal {D}})$ denotes the topological dual space of $H_0^\eta ({\mathcal {D}})$. Moreover, if the ${\mathcal {X}}$ we are considering is itself a Bochner or Sobolev–Bochner space, then we replace the ${\mathcal {X}}$ in the subscript of the norm with the subscripts of its norm, for example

$$\begin{aligned} \big \Vert v\big \Vert _{p, {\mathcal {M}}; \eta , q, {\mathcal {D}}; {\mathcal {Y}}} = \big \Vert v\big \Vert _{p, {\mathcal {M}}; W^{\eta , q}({\mathcal {D}}; {\mathcal {Y}})} = \big \Vert v\big \Vert _{L_\mu ^p({\mathcal {M}}; W^{\eta , q}({\mathcal {D}}; {\mathcal {Y}}))} . \end{aligned}$$

Further, for computational complexity estimates we will make use of the Big Theta notation, that is $f = \varTheta (g)$ means that $f = {\mathcal {O}}(g)$ and $g = {\mathcal {O}}(f)$. Lastly, to avoid the use of generic but unspecified constants in certain formulas, we use $c \lesssim d$ to mean that *c* can be bounded by a multiple of *d*, independently of parameters which *c* and *d* may depend on. Obviously, $c > rsim d$ is defined as $d \lesssim c$ and we write $c \eqsim d$ if $c \lesssim d$ and $c > rsim d$.

### Model problem

Let $\tau \in {\mathbb {N}}$ and $d \in {\mathbb {N}}^*$; $D \subset {\mathbb {R}}^d$ denote the *reference domain* with boundary $\partial D$ that is of class $C^{\tau +1}$—when $\tau = 1$ then we also consider the case where *D* is a bounded and convex domain with Lipschitz continuous boundary—and $(\varOmega , {\mathcal {F}}, {\mathbb {P}})$ be a separable, complete probability space with $\sigma$-field ${\mathcal {F}}\subset 2^\varOmega$ and probability measure ${\mathbb {P}}$. Furthermore, let

$$\begin{aligned} {\mathbf {V}}\in L_{\mathbb {P}}^\infty \big (\varOmega ; C^{\tau , 1}({\overline{D}}; {\mathbb {R}}^d)\big ) \end{aligned}$$

be the *random domain mapping*. Moreover, we require that, for ${\mathbb {P}}$-almost any $\omega$, ${\mathbf {V}}[\omega ] :D \rightarrow {\mathfrak {D}}[\omega ]$ is bi-Lipschitz and fulfils the *uniformity condition*

$$\begin{aligned} {\underline{\sigma }} \le \big |{\mathbf {V}}[\omega ]\big |_{{\underline{Lip}}(D; {\mathbb {R}}^d)} \le \big |{\mathbf {V}}[\omega ]\big |_{{\overline{Lip}}(D; {\mathbb {R}}^d)} \le {\overline{\sigma }} \end{aligned}$$

for $0< {\underline{\sigma }} \le {\overline{\sigma }} < \infty$ independent of $\omega$. Finally, we require that the we have a *hold-all domain* ${\mathcal {D}}$ that satisfies ${\mathfrak {D}}[\omega ] \subset {\mathcal {D}}$ for ${\mathbb {P}}$-almost any $\omega \in \varOmega$, a *deterministic subdomain* *B* that satisfies ${{\,\mathrm{dist}\,}}\big \{B, \partial {\mathfrak {D}}[\omega ]\big \} > \delta$ for ${\mathbb {P}}$-almost any $\omega \in \varOmega$ with a $\delta > 0$ and consider $f \in C^\omega ({\mathcal {D}})$.

While, by definition, we know that ${\mathbf {V}}[\omega ]$ is a $C^{0,1}$-diffeomorphism from $D \rightarrow {\mathfrak {D}}[\omega ]$ for ${\mathbb {P}}$-almost any $\omega \in \varOmega$, we also have the following stronger result.

#### Proposition 1

For ${\mathbb {P}}$-almost any $\omega \in \varOmega$, ${\mathbf {V}}[\omega ]$ is a $C^{\tau ,1}$-diffeomorphism from *D* to ${\mathfrak {D}}[\omega ]$.

#### Proof

The fact that ${\mathbf {V}}[\omega ]$ is a $C^{\tau }$-diffeomorphism follows directly from the inverse funtion theorem. Then, with the explicit formula for the $\tau$-th derivative of ${\mathbf {V}}[\omega ]^{-1}$ from the inverse funtion theorem, one can bound $\big |{{\,\mathrm{D}\,}}^{\tau } {\mathbf {V}}[\omega ]^{-1}\big |_{{\overline{Lip}}(D; {\mathbb {R}}^d)}$ independently of $\omega$. $\square$

Now, since for ${\mathbb {P}}$-almost any $\omega \in \varOmega$ we have a $C^{\tau ,1}$-diffeomorphism from $D \rightarrow {\mathfrak {D}}[\omega ]$ we can use the one-to-one correspondence to pull back the model problem onto the reference domain *D* instead of considering it on the actual domain realisations ${\mathfrak {D}}[\omega ]$. According to the chain rule, we then have for $v \in H^1({\mathfrak {D}}[\omega ])$ that $v \circ {\mathbf {V}}[\omega ] \in H^1(D)$ and

$$\begin{aligned} (\nabla _{\mathbf {x}}v) \circ {\mathbf {V}}[\omega ] = \big ({\mathbf {J}}[\omega ]\big )^{-{\mathsf {T}}} \nabla _{\mathbf {x}}\big (v \circ {\mathbf {V}}[\omega ]\big ) . \end{aligned}$$

Now, with (3) this leads us to the following formulation of our model problem (2) on the reference domain, cf. [28]:

6 $$\begin{aligned} \left\{ \begin{aligned}&\text {Find }{\hat{u}} \in L_{\mathbb {P}}^\infty \big (\varOmega ; H_0^1(D)\big )\text { such that} \\&\qquad \int _{D} \big \langle {\hat{{\mathbf {A}}}}[\omega ]({\mathbf {x}}) \nabla _{\mathbf {x}}{\hat{u}}[\omega ]({\mathbf {x}}), \nabla _{\mathbf {x}}{\hat{v}}({\mathbf {x}})\big \rangle {{\,\mathrm{d}\,}}\!{\mathbf {x}}= \int _{D} {\hat{f}}[\omega ]({\mathbf {x}}) {\hat{v}}({\mathbf {x}}) {{\,\mathrm{d}\,}}\!{\mathbf {x}}\\&\text {for }{\mathbb {P}}\text {-almost every }\omega \in \varOmega \text { and all } {\hat{v}} \in H_0^1(D). \end{aligned} \right. \end{aligned}$$

Note, especially, that by the uniformity condition we have that

7 $$\begin{aligned} \frac{{\underline{\sigma }}^d}{{\overline{\sigma }}^2} \le \mathop {\hbox {ess inf}}\limits _{\omega \in \varOmega } \mathop {\hbox {ess inf}}\limits _{{\mathbf {x}}\in D} \lambda _{\min } \big ({\hat{{\mathbf {A}}}}[\omega ]({\mathbf {x}})\big ) \le \mathop {\hbox {ess sup}}\limits _{\omega \in \varOmega } \mathop {\hbox {ess sup}}\limits _{{\mathbf {x}}\in D} \lambda _{\max } \big ({\hat{{\mathbf {A}}}}[\omega ]({\mathbf {x}})\big ) \le \frac{{\overline{\sigma }}^d}{{\underline{\sigma }}^2} . \end{aligned}$$

Without loss of generality, we assume ${\underline{\sigma }} \le 1 \le {\overline{\sigma }}$.

From here on, we assume that the spatial variable ${\mathbf {x}}$ and the stochastic parameter $\omega$ of the random field have been separated by the Karhunen–Loève expansion of ${\mathbf {V}}$ coming from the mean field ${\mathbb {E}}[{\mathbf {V}}]$ and the covariance ${\mathbb {C}}\mathrm{ov}[{\mathbf {V}}]$ yielding a parametrised expansion

8 $$\begin{aligned} {\mathbf {V}}[{\mathbf {y}}]({\mathbf {x}}) = {\mathbb {E}}[{\mathbf {V}}]({\mathbf {x}}) + \sum _{k=1}^{\infty } \sigma _k {\varvec{\psi }}_k({\mathbf {x}}) y_k , \end{aligned}$$

where ${\mathbf {y}}= (y_k)_{k \in {\mathbb {N}}^*} \in {\mathbb {R}}^{{\mathbb {N}}^*}$ is a sequence of uncorrelated random variables, see e.g. [28]. We now impose some common assumptions, which make the Karhunen–Loève expansion computationally feasible.

#### Assumption 1

1. The random variables $(y_k)_{k \in {\mathbb {N}}^*}$ are independent and identically distributed. Moreover, they are uniformly distributed on $\left[ {{-\frac{1}{2}}, \frac{1}{2}}\right]$.
2. We assume that the ${\varvec{\psi }}_k$ are elements of $C^{\tau ,1}({\overline{D}}; {\mathbb {R}}^d)$ and that the sequence ${\varvec{\gamma }}= \big (\gamma _k\big )_{k \in {\mathbb {N}}}$, given by
  $$\begin{aligned} \gamma _k \mathrel {\mathrel {\mathop :}=}\big \Vert \sigma _k {\varvec{\psi }}_k\big \Vert _{C^{\tau ,1}({\overline{D}}; {\mathbb {R}}^d)} , \end{aligned}$$
  is at least in $\ell ^1({\mathbb {N}})$, where we have defined ${\varvec{\psi }}_0 \mathrel {\mathrel {\mathop :}=}{\mathbb {E}}[{\mathbf {V}}]$ and $\sigma _0 \mathrel {\mathrel {\mathop :}=}1$. Furthermore, we define
  $$\begin{aligned} c_{{\varvec{\gamma }}} = \max \big \{ {\big \Vert {\varvec{\gamma }}\big \Vert _{\ell ^1({\mathbb {N}})}, 1\big \}} . \end{aligned}$$

Therefore, we now can restrict ${\mathbf {y}}$ to be in $\square \mathrel {\mathrel {\mathop :}=}\left[ {{-\frac{1}{2}}, \frac{1}{2}}\right] ^{{\mathbb {N}}^*}$ and introduce the pushforward measure of ${\mathbb {P}}$ onto $\square$ as ${\mathbb {P}}_{\mathbf {y}}$. We then view all randomness as being parametrised by ${\mathbf {y}}$, i.e. from the next section onwards $\omega$, $\varOmega$ and ${\mathbb {P}}$ are considered to have been replaced by ${\mathbf {y}}$, $\square$ and ${\mathbb {P}}_{\mathbf {y}}$.

#### Remark 1

Note that while we restrict ourselves to the stated model problem here to simplify the analysis, the regularity result can be extended. For example, it is not necessary that ${\mathbf {V}}$ has an affine dependence on ${\mathbf {y}}$ as in (8), as long as a weakend version of Lemma 2 with bounds of the form $\big |{\varvec{\alpha }}\big |! k_{{\mathbf {V}}{\mathbf {J}}} c_{{\mathbf {V}}{\mathbf {J}}}^{|{{\varvec{\alpha }}}|} {\varvec{\gamma }}^{\varvec{\alpha }}$ stays true. Moreover, it is also possible to consider the partial differential equation

$$\begin{aligned} -{{\,\mathrm{div}\,}}_{\mathbf {x}}{\mathbf {A}}({\mathbf {x}}) \nabla _{\mathbf {x}}u[\omega ] = f \text { in } {\mathfrak {D}}[\omega ], \end{aligned}$$

instead of the one in (1) for an ${\mathbf {A}}\in C^\omega ({\mathcal {D}}; {\mathbb {R}}^{d \times d}_{\mathrm {symm}})$ with ${\mathbf {A}}$ fulfilling an ellipticity condition, that is to prescribe a deterministic diffusion coefficient in Eulerian coordinates; or to consider

$$\begin{aligned} -{{\,\mathrm{div}\,}}_{\mathbf {x}}{\mathbf {A}}[\omega ]\big ({\mathbf {V}}[\omega ]^{-1}({\mathbf {x}})\big ) \nabla _{\mathbf {x}}u[\omega ] = f[\omega ]\big ({\mathbf {V}}[\omega ]^{-1}({\mathbf {x}})\big ) \text { in } {\mathfrak {D}}[\omega ], \end{aligned}$$

for an ${\mathbf {A}}\in L_{\mathbb {P}}^\infty \big (\varOmega ; C^{\tau -1, 1}({\overline{D}}; {\mathbb {R}}^{d \times d}_{\mathrm {symm}})\big )$ and $f \in L_{\mathbb {P}}^\infty \big (\varOmega ; H^{\tau -1}(D)\big )$ with ${\mathbf {A}}$ fulfilling an ellipticity condition almost surely almost everywhere, that is to prescribe a stochastic diffusion coefficient and loading in Lagrangian coordinates.

## Regularity

Our aim is to consider quantities of interest that are of the form

$$\begin{aligned} {{\,\mathrm{QoI}\,}}(u) = \int _\square {\mathcal {F}}\big (u[{\mathbf {y}}]\vert _B\big ) {{\,\mathrm{d}\,}}\!{\mathbb {P}}_{\mathbf {y}}, \end{aligned}$$

where ${\mathcal {F}}$ is a smooth operator into a Banach space ${\mathcal {X}}$, that is ${\mathcal {F}}:H^\sigma (B) \rightarrow {\mathcal {X}}$ is analytic for $\sigma \le \tau$. However, since we will require our domain mapping to fulfil^5^${\mathbf {V}}[{\mathbf {y}}]\vert _B = {{\,\mathrm{Id}\,}}_B$, we will be able to use the fact that ${\hat{u}}[{\mathbf {y}}]\vert _B = u[{\mathbf {y}}]\vert _B$. Therefore, we now discuss the regularity of the mapping ${\hat{u}} :\square \rightarrow H^{\tau +1}(D)$, as that then directly implies the regularity of the mapping $u\vert _B :\square \rightarrow H^{\tau +1}(B)$. Showing that the mapping is analytic justifies considering many discretisations for the computation of the integral. However, having that smoothness with regard to the space $H^{\tau +1}(B)$ instead of only $H^{\sigma }(B)$ justifies the use of their respective multilevel version, see for example [27].

To prove the analyticity of the mapping ${\hat{u}} :\square \rightarrow H^{\tau +1}(D)$, we first investigate the analyticity of the mappings ${\hat{{\mathbf {A}}}} :\square \rightarrow W^{\tau , \infty }(D; {\mathbb {R}}^{d \times d}_{\mathrm {symm}})$ and ${\hat{f}} :\square \rightarrow H^{\tau -1}(D)$. Based on that analyticity we then can essentially leverage results from [30] to arrive at the analyticity for ${\hat{u}}$. Indeed, the whole section relies heavily on the regularity results from [30] and uses the same notations: Note especially, that the weighting in the Sobolev–Bochner norms makes them submultiplicative and that to make the notation less cumbersome, since we are considering the norm of spaces of the form $L_{{\mathbb {P}}_{\mathbf {y}}}^\infty \big (\square ; {\mathcal {X}}\big )$, we use the shorthand notation

$$\begin{aligned} \big |\big |\big |v\big |\big |\big |_{{\mathcal {X}}} \mathrel {\mathrel {\mathop :}=}\big \Vert v\big \Vert _{\infty , \square ; {\mathcal {X}}} . \end{aligned}$$

As we mainly make use it for spaces of the form $L_{{\mathbb {P}}_{\mathbf {y}}}^\infty \big (\square ; W^{\eta , p}(D; {\mathcal {X}})\big )$, this then becomes $\big |\big |\big |v\big |\big |\big |_{\eta , p, D; {\mathcal {X}}} = \big \Vert v\big \Vert _{\infty , \square ; \eta , p, D; {\mathcal {X}}}$.

### A combinatorial lemma

In the following subsection we will derive the bounds on the derivatives of the diffusion coefficient and the loading piece by piece by using addition, multiplication and composition of functions with bounds of the form

$$\begin{aligned} \big \Vert {{\,\mathrm{D}\,}}^r \cdot \big \Vert \le r! k c^r \end{aligned}$$

and using [30, Lemma 2, 3 and 4]. To be able to combine bounds on the derivatives of functions combined by composition with the bounds of the inner function being of the form

$$\begin{aligned} \big \Vert \partial ^{{\varvec{\alpha }}} \cdot \big \Vert \le \big |{\varvec{\alpha }}\big |! k c^{|{{\varvec{\alpha }}}|} {\varvec{\gamma }}^{{\varvec{\alpha }}} \end{aligned}$$

we will use [30, Lemma 8] together with the following combinatorial lemma.

#### Lemma 1

Let ${\varvec{\alpha }}\in {\mathbb {N}}^{{\mathbb {N}}^*}_f$ be a multi–index with ${\varvec{\alpha }}\ne {{\varvec{0}}}$ and $r \in {\mathbb {N}}^*$ with $r \le \big |{\varvec{\alpha }}\big |$. Then, we have

$$\begin{aligned} {\varvec{\alpha }}! \sum _{C({\varvec{\alpha }}, r)} \prod _{j=1}^{r} \frac{\big |{\varvec{\beta }}_j\big |!}{{\varvec{\beta }}_j!} = [{{\varvec{\alpha }}}]! \left( {\begin{array}{c}\big |{\varvec{\alpha }}\big | - 1\\ r - 1\end{array}}\right) . \end{aligned}$$

where $C({\varvec{\alpha }}, r)$ is the set of all compositions of the multi-index ${\varvec{\alpha }}$ into *r* non-vanishing multi-indices ${\varvec{\beta }}_1, \ldots , {\varvec{\beta }}_r$,

$$\begin{aligned} C({\varvec{\alpha }}, r) \mathrel {\mathrel {\mathop :}=}\bigg \{ \big ({\varvec{\beta }}_1, \ldots , {\varvec{\beta }}_r\big ) \in \big ({\mathbb {N}}^{{\mathbb {N}}^*}_f\big )^r : \sum _{j=1}^r {\varvec{\beta }}_j = {\varvec{\alpha }}\text { and } {\varvec{\beta }}_{j} \ne {{\varvec{0}}}\text { for all } 1 \le j \le r \bigg \} . \end{aligned}$$

#### Proof

For convenience, we introduce the following notation for this proof: For a multi-index ${\varvec{\beta }}\in {\mathbb {N}}^{{\mathbb {N}}^*}_f$ with ${\varvec{\beta }}\ne {{\varvec{0}}}$ we say that ${\mathbf {s}}\in {\mathbb {N}}^{|{{\varvec{\beta }}}|}$ is a serialisation of ${\varvec{\beta }}$ if for any $n \in {{\,\mathrm{supp}\,}}{\varvec{\beta }}$ there exist exactly $\beta _n$ different $j \in \big \{1, \ldots , \big |{\varvec{\beta }}\big |\big \}$ such that $s_j = n$.

Now, as the expression

$$\begin{aligned} \frac{\big |{\varvec{\beta }}\big |!}{{\varvec{\beta }}!} \end{aligned}$$

is just a compact notation for the multinomial, it is equal to the cardinality of the set containing all serialisations of ${\varvec{\beta }}$. Therefore, for any $\big ({\varvec{\beta }}_1, \ldots , {\varvec{\beta }}_r\big ) \in C({\varvec{\alpha }}, r)$,

$$\begin{aligned} \prod _{j=1}^{r} \frac{\big |{\varvec{\beta }}_j\big |!}{{\varvec{\beta }}_j!} \end{aligned}$$

is the cardinality of the set

$$\begin{aligned} \bigg \{ \big ({\mathbf {s}}_1, \ldots , {\mathbf {s}}_r\big ) \in {\mathbb {N}}{^{|{\varvec{\beta }}_1|}} \times \cdots \times {\mathbb {N}}{^{|{\varvec{\beta }}_r|}} : {\mathbf {s}}_j \text { is a serialisation of } {\varvec{\beta }}_{j} \text { for all } 1 \le j \le r \bigg \} . \end{aligned}$$

Thus, the expression

$$\begin{aligned} \sum _{C({\varvec{\alpha }}, r)} \prod _{j=1}^{r} \frac{\big |{\varvec{\beta }}_j\big |!}{{\varvec{\beta }}_j!} \end{aligned}$$

gives the cardinality of the set

$$\begin{aligned}&\bigg \{ \big ({\mathbf {s}}_1, \ldots , {\mathbf {s}}_r\big ) \in {\mathbb {N}}^{k_1} \times \cdots \times {\mathbb {N}}^{k_r} : \sum _{j=1}^{r} k_j = \big |{\varvec{\alpha }}\big | \text { and } k_j \in {\mathbb {N}}^* \text { for all } 1 \le j \le r \\&\text { and the concatenation of the } {\mathbf {s}}_j \text { is a serialisation of } {\varvec{\alpha }}\bigg \} , \end{aligned}$$

which may also be seen as the set giving all the ways to cut all the serialisations of ${\varvec{\alpha }}$ into *r* non-empty blocks. The cardinality is thus also given by the expression

$$\begin{aligned} \frac{\big |{\varvec{\alpha }}\big |!}{{\varvec{\alpha }}!} \left( {\begin{array}{c}\big |{\varvec{\alpha }}\big | - 1\\ r - 1\end{array}}\right) , \end{aligned}$$

as the first factor counts the serialisations of ${\varvec{\alpha }}$ and the second the ways to cut a sequence of length $\big |{\varvec{\alpha }}\big |$ into *r* non-empty blocks, which yields the desired assertion

$$\begin{aligned} \sum _{C({\varvec{\alpha }}, r)} \prod _{j=1}^{r} \frac{\big |{\varvec{\beta }}_j\big |!}{{\varvec{\beta }}_j!} = \frac{\big |{\varvec{\alpha }}\big |!}{{\varvec{\alpha }}!} \left( {\begin{array}{c}\big |{\varvec{\alpha }}\big | - 1\\ r - 1\end{array}}\right) . \end{aligned}$$

$$\square $$

#### Remark 2

We will use this combinatorial lemma to give the following bound

9 $$\begin{aligned} {\varvec{\alpha }}! \sum _{C({\varvec{\alpha }}, r)} \prod _{j=1}^{r} \frac{1}{{\varvec{\beta }}_j!} \le {\varvec{\alpha }}! \sum _{C({\varvec{\alpha }}, r)} \prod _{j=1}^{r} \frac{\big |{\varvec{\beta }}_j\big |!}{{\varvec{\beta }}_j!} = \big |{\varvec{\alpha }}\big |! \left( {\begin{array}{c}\big |{\varvec{\alpha }}\big | - 1\\ r - 1\end{array}}\right) . \end{aligned}$$

We note that this bound can be improved by using the identity

$$\begin{aligned} {\varvec{\alpha }}! \sum _{C({\varvec{\alpha }}, r)} \prod _{j=1}^{r} \frac{1}{{\varvec{\beta }}_j!} = r! S_{|{{\varvec{\alpha }}}|, r} \end{aligned}$$

with $S_{n, r}$ denoting the Stirling numbers of the second kind and bounding this, as is done, for example, in [30], which will yield smaller constants $k_{{\hat{{\mathbf {A}}}}}, c_{{\hat{{\mathbf {A}}}}}, k_{{\hat{f}}}, c_{{\hat{f}}}$ in Theorems 1 and 2. However, using this identity is more restrictive as it requires Lemma 2 to hold as stated, whereas, by the bound (9), we actually only require a weakend version of Lemma 2, as noted in Remark 1.

### Parametric regularity of the diffusion coefficient and the loading

To provide regularity estimates for the diffusion coefficient ${\hat{{\mathbf {A}}}}$ and the right hand side ${\hat{f}}$, that are based on the decay of the expansion of ${\mathbf {V}}$ as per Assumption 1, we first note that we can write^6^

10 $$\begin{aligned} {\hat{{\mathbf {A}}}}[{\mathbf {y}}]({\mathbf {x}}) = {\mathbf {T}}\big ({\mathbf {V}}[{\mathbf {y}}]({\mathbf {x}}), {\mathbf {J}}[{\mathbf {y}}]({\mathbf {x}})\big ) \quad \text {and}\quad {\hat{f}}[{\mathbf {y}}]({\mathbf {x}}) = s\big ({\mathbf {V}}[{\mathbf {y}}]({\mathbf {x}}), {\mathbf {J}}[{\mathbf {y}}]({\mathbf {x}})\big ) \end{aligned}$$

with

11 $$\begin{aligned}&{\mathbf {T}}:{\mathcal {D}}\times {\mathbb {R}}^{d \times d}_{{\underline{\sigma }}, {\overline{\sigma }}} \rightarrow {\mathbb {R}}^{d \times d}_{\mathrm {symm}} ,\, ({\mathbf {x}}, {\mathbf {M}}) \mapsto ({\mathbf {M}}^{\mathsf {T}}{\mathbf {M}})^{-1} \det {\mathbf {M}}\end{aligned}$$

12 $$\begin{aligned}&s :{\mathcal {D}}\times {\mathbb {R}}^{d \times d}_{{\underline{\sigma }}, {\overline{\sigma }}} \rightarrow {\mathbb {R}},\, ({\mathbf {x}}, {\mathbf {M}}) \mapsto f({\mathbf {x}}) \det {\mathbf {M}}, \end{aligned}$$

where ${\mathbb {R}}^{d \times d}_{{\underline{\sigma }}, {\overline{\sigma }}} \mathrel {\mathrel {\mathop :}=}\bigg \{{\mathbf {M}}\in {\mathbb {R}}^{d \times d} \,:\, {\underline{\sigma }} \le \sigma _{\min }({\mathbf {M}}) \le \sigma _{\max }({\mathbf {M}}) \le {\overline{\sigma }}\bigg \}$. Therefore, we first discuss the regularity of the combined mapping

$$\begin{aligned} ({\mathbf {V}}, {\mathbf {J}}) :\square \rightarrow \big (D \rightarrow {\mathcal {D}}\times {\mathbb {R}}^{d \times d}_{{\underline{\sigma }}, {\overline{\sigma }}}\big ) ,\, {\mathbf {y}}\mapsto \big ({\mathbf {x}}\mapsto \big ({\mathbf {V}}[{\mathbf {y}}]({\mathbf {x}}), {\mathbf {J}}[{\mathbf {y}}]({\mathbf {x}})\big )\big ) , \end{aligned}$$

for which we have the following result.^7^

#### Lemma 2

We have for all ${\varvec{\alpha }}\in {\mathbb {N}}^{{\mathbb {N}}^*}_f$ that

$$\begin{aligned} \big |\big |\big |\partial _{\mathbf {y}}^{\varvec{\alpha }}({\mathbf {V}},{\mathbf {J}})\big |\big |\big |_{\tau , \infty , D} \le k_{{\mathbf {V}}{\mathbf {J}}} {\varvec{\gamma }}^{\varvec{\alpha }}, \end{aligned}$$

where $k_{{\mathbf {V}}{\mathbf {J}}} \mathrel {\mathrel {\mathop :}=}[1 + (\tau +1) d] c_{\tau } c_{{\varvec{\gamma }}}$. Here, $c_{\tau }$ denotes the constant coming from the embedding $C^{\tau , 1}({\overline{D}}; {\mathbb {R}}^d) \hookrightarrow W^{\tau +1, \infty }(D; {\mathbb {R}}^d)$.

#### Proof

By definition we have that ${\mathbf {J}}[{\mathbf {y}}] = {{\,\mathrm{D}\,}}_{\mathbf {x}}{\mathbf {V}}[{\mathbf {y}}]$ and so it follows that

$$\begin{aligned} {\mathbf {V}}[{\mathbf {y}}] = \sigma _0 {\varvec{\psi }}_0 + \sum _{k=1}^{\infty } \sigma _k {\varvec{\psi }}_k y_k \quad \text {and}\quad {\mathbf {J}}[{\mathbf {y}}] = \sigma _0 {{\,\mathrm{D}\,}}_{\mathbf {x}}{\varvec{\psi }}_0 + \sum _{k=1}^{\infty } \sigma _k {{\,\mathrm{D}\,}}_{\mathbf {x}}{\varvec{\psi }}_k y_k . \end{aligned}$$

From this we can derive that first order derivatives are given by

$$\begin{aligned} \partial _{y_i}{\mathbf {V}}[{\mathbf {y}}] = \sigma _i {\varvec{\psi }}_i \quad \text {and}\quad \partial _{y_i}{\mathbf {J}}[{\mathbf {y}}] = \sigma _i {{\,\mathrm{D}\,}}_{\mathbf {x}}{\varvec{\psi }}_i \end{aligned}$$

for $i \in {\mathbb {N}}^*$ and all higher derivatives vanish. Clearly, this affine dependence on ${\mathbf {y}}$ implies the bounds. $\square$

Next, we supply bounds on the derivatives of the mappings ${\mathbf {T}}$ and *s*.

#### Lemma 3

The mapping ${\mathbf {T}}$ is infinitely Fréchet differentiable with

$$\begin{aligned} \big \Vert {{\,\mathrm{D}\,}}^r {\mathbf {T}}({\mathbf {x}}, {\mathbf {M}})\big \Vert _{{\mathcal {B}}^r({\mathbb {R}}^d \times {\mathbb {R}}^{d \times d}; {\mathbb {R}}^{d \times d}_{\mathrm {symm}})} \le r! k_{\mathbf {T}}c_{\mathbf {T}}^r \end{aligned}$$

for all $r \in {\mathbb {N}}$ and $({\mathbf {x}}, {\mathbf {M}}) \in {\mathcal {D}}\times {\mathbb {R}}^{d \times d}_{{\underline{\sigma }}, {\overline{\sigma }}}$ with $k_{\mathbf {T}}= {\underline{\sigma }}^{-2} (2 {\overline{\sigma }})^d$ and $c_{\mathbf {T}}= 4 ({\underline{\sigma }}^{-2} {\overline{\sigma }}^2 + 1)$.

#### Proof

We start with the mappings

$$\begin{aligned} {\mathbf {T}}_1&:{\mathcal {D}}\times {\mathbb {R}}^{d \times d}_{{\underline{\sigma }}, {\overline{\sigma }}} \rightarrow {\mathbb {R}}^{d \times d}_{{\underline{\sigma }}, {\overline{\sigma }}} ,\, ({\mathbf {x}}, {\mathbf {M}}) \mapsto {\mathbf {M}}\quad \text {and} \\ {\mathbf {T}}_2&:{\mathcal {D}}\times {\mathbb {R}}^{d \times d}_{{\underline{\sigma }}, {\overline{\sigma }}} \rightarrow {\mathbb {R}}^{d \times d}_{{\underline{\sigma }}, {\overline{\sigma }}} ,\, ({\mathbf {x}}, {\mathbf {M}}) \mapsto {\mathbf {M}}^{\mathsf {T}}, \end{aligned}$$

which are infinitely Fréchet differentiable with

$$\begin{aligned} \big \Vert {{\,\mathrm{D}\,}}^r {\mathbf {T}}_i({\mathbf {x}}, {\mathbf {M}})\big \Vert _{{\mathcal {B}}^r({\mathbb {R}}^d \times {\mathbb {R}}^{d \times d}; {\mathbb {R}}^{d \times d})} \le r! k_i c_i^r \end{aligned}$$

for all $({\mathbf {x}}, {\mathbf {M}}) \in {\mathcal {D}}\times {\mathbb {R}}^{d \times d}_{{\underline{\sigma }}, {\overline{\sigma }}}$, $i = 1, 2$ and $k_1 = k_2 = {\overline{\sigma }}$, $c_1 = c_2 = 1$. Then, using [30, Lemma 3], we see that the mapping

$$\begin{aligned} {\mathbf {T}}_3 :{\mathcal {D}}\times {\mathbb {R}}^{d \times d}_{{\underline{\sigma }}, {\overline{\sigma }}} \rightarrow {\mathbb {R}}^{d \times d}_{{\underline{\sigma }}^2, {\overline{\sigma }}^2} ,\, ({\mathbf {x}}, {\mathbf {M}}) \mapsto {\mathbf {M}}^{\mathsf {T}}{\mathbf {M}}\end{aligned}$$

is infinitely Fréchet differentiable with

$$\begin{aligned} \big \Vert {{\,\mathrm{D}\,}}^r {\mathbf {T}}_3({\mathbf {x}}, {\mathbf {M}})\big \Vert _{{\mathcal {B}}^r({\mathbb {R}}^d \times {\mathbb {R}}^{d \times d}; {\mathbb {R}}^{d \times d})} \le r! k_3 c_3^r \end{aligned}$$

for all $({\mathbf {x}}, {\mathbf {M}}) \in {\mathcal {D}}\times {\mathbb {R}}^{d \times d}_{{\underline{\sigma }}, {\overline{\sigma }}}$, and $k_3 = {\overline{\sigma }}^2$, $c_3 = 2$.

Next, we consider the mapping

$$\begin{aligned} \mathbf {inv}:{\mathbb {R}}^{d \times d}_{{\underline{\sigma }}^2, {\overline{\sigma }}^2} \rightarrow {\mathbb {R}}^{d \times d}_{{\overline{\sigma }}^{-2}, {\underline{\sigma }}^{-2}} ,\, {\mathbf {M}}\mapsto {\mathbf {M}}^{-1} . \end{aligned}$$

Clearly, the *r*-th Fréchet derivative of $\mathbf {inv}$ at the point ${\mathbf {M}}\in {\mathbb {R}}^{d \times d}_{{\underline{\sigma }}^2, {\overline{\sigma }}^2}$ in the directions of ${\mathbf {H}}_1, \ldots , {\mathbf {H}}_r \in {\mathbb {R}}^{d \times d}$ is given by

$$\begin{aligned} {{\,\mathrm{D}\,}}^r \mathbf {inv}({\mathbf {M}}){\mathbf {H}}_1 \cdots {\mathbf {H}}_r&= (-1)^r \sum _{\sigma \in S_r} {\mathbf {M}}^{-1} \prod _{j=1}^{r} \big ({\mathbf {H}}_{\sigma (j)} {\mathbf {M}}^{-1}\big )\\&= (-1)^r \sum _{\sigma \in S_r} \mathbf {inv}({\mathbf {M}}) \prod _{j=1}^{r} \big ({\mathbf {H}}_{\sigma (j)} \mathbf {inv}({\mathbf {M}})\big ) , \end{aligned}$$

where $S_r$ is the set of all bijections on the set $\big \{1, 2, \cdots , r\big \}$. Thus, we have

$$\begin{aligned} \big \Vert {{\,\mathrm{D}\,}}^r \mathbf {inv}({\mathbf {M}})\big \Vert _{{\mathcal {B}}^{r}({\mathbb {R}}^{d \times d}; {\mathbb {R}}^{d \times d})} \le r! \big \Vert \mathbf {inv}({\mathbf {M}})\big \Vert _2^{r+1} \le r! k_{\mathbf {inv}} c_{\mathbf {inv}}^r \end{aligned}$$

for all ${\mathbf {M}}\in {\mathbb {R}}^{d \times d}_{{\underline{\sigma }}^2, {\overline{\sigma }}^2} \rightarrow {\mathbb {R}}^{d \times d}_{{\overline{\sigma }}^{-2}, {\underline{\sigma }}^{-2}}$ with $k_{\mathbf {inv}} = c_{\mathbf {inv}} = {\underline{\sigma }}^{-2}$. Therefore, we can use [30, Lemma 4] to see that the mapping

$$\begin{aligned} {\mathbf {T}}_4 :{\mathcal {D}}\times {\mathbb {R}}^{d \times d}_{{\underline{\sigma }}, {\overline{\sigma }}} \rightarrow {\mathbb {R}}^{d \times d}_{{\overline{\sigma }}^{-2}, {\underline{\sigma }}^{-2}} ,\, ({\mathbf {x}}, {\mathbf {M}}) \mapsto \mathbf {inv}\big ({\mathbf {T}}_3({\mathbf {x}}, {\mathbf {M}})\big ) = ({\mathbf {M}}^{\mathsf {T}}{\mathbf {M}})^{-1} \end{aligned}$$

is infinitely Fréchet differentiable with

$$\begin{aligned} \big \Vert {{\,\mathrm{D}\,}}^r {\mathbf {T}}_4({\mathbf {x}}, {\mathbf {M}})\big \Vert _{{\mathcal {B}}^r({\mathbb {R}}^d \times {\mathbb {R}}^{d \times d}; {\mathbb {R}}^{d \times d})} \le r! k_4 c_4^r \end{aligned}$$

for all $({\mathbf {x}}, {\mathbf {M}}) \in {\mathcal {D}}\times {\mathbb {R}}^{d \times d}_{{\underline{\sigma }}, {\overline{\sigma }}}$, and $k_4 = {\underline{\sigma }}^{-2}$, $c_4 = ({\underline{\sigma }}^{-2} {\overline{\sigma }}^2 + 1) 2$.

Finally, we consider the mapping

$$\begin{aligned} {{\,\mathrm{det}\,}}:{\mathbb {R}}^{d \times d}_{{\underline{\sigma }}, {\overline{\sigma }}} \rightarrow {\mathbb {R}},\, {\mathbf {M}}\mapsto {{\,\mathrm{det}\,}}{\mathbf {M}}, \end{aligned}$$

which has the *r*-th Fréchet derivative of ${{\,\mathrm{det}\,}}$ given by^8^

$$\begin{aligned} {{\,\mathrm{D}\,}}^r {{\,\mathrm{det}\,}}({\mathbf {M}}){\mathbf {H}}_1 \cdots {\mathbf {H}}_r = \sum _{\begin{array}{c} 1 \le i_1, \ldots , i_r \le d\\ \text {p.w. inequal} \end{array}} \det \big ({\mathbf {M}}_{[i_1, {\mathbf {H}}_1], \ldots , [i_r, {\mathbf {H}}_r]}\big ) , \end{aligned}$$

where ${\mathbf {M}}_{[i_1, {\mathbf {H}}_1], \ldots , [i_r, {\mathbf {H}}_r]}$ denotes the matrix ${\mathbf {M}}$ whose $i_k$-th column is replaced by the $i_k$-th column of the matrix ${\mathbf {H}}_k$ for all *k* from 1 to *r*. Now, since we can bound the determinant of a matrix by the product of the norms of its columns, i.e.

$$\begin{aligned} \bigg |{{\,\mathrm{det}\,}}\left( {\begin{bmatrix} {\mathbf {z}}_1&\cdots&{\mathbf {z}}_d\end{bmatrix}}\right) \bigg | \le \prod _{j=1}^{d} \big \Vert {\mathbf {z}}_j\big \Vert , \end{aligned}$$

and since we know that

$$\begin{aligned} \big \Vert {\mathbf {z}}_j\big \Vert \le \big \Vert \begin{bmatrix} {\mathbf {z}}_1&\cdots&{\mathbf {z}}_d\end{bmatrix}\big \Vert . \end{aligned}$$

it follows that,

$$\begin{aligned} \big \Vert {{\,\mathrm{D}\,}}^r {{\,\mathrm{det}\,}}({\mathbf {M}})\big \Vert _{{\mathcal {B}}^{r}({\mathbb {R}}^{d \times d}; {\mathbb {R}})} \le \frac{d!}{(d-r)!} \big \Vert {\mathbf {M}}\big \Vert ^{d-r} \le r! \left( {\begin{array}{c}d\\ r\end{array}}\right) {\overline{\sigma }}^d \le r! k_{{{\,\mathrm{det}\,}}} c_{{{\,\mathrm{det}\,}}}^r , \end{aligned}$$

with $k_{{{\,\mathrm{det}\,}}} = (2 {\overline{\sigma }})^d$ and $c_{{{\,\mathrm{det}\,}}} = 1$. As before, we can use [30, Lemma 4] to see that the mapping

$$\begin{aligned} T_5 :{\mathcal {D}}\times {\mathbb {R}}^{d \times d}_{{\underline{\sigma }}, {\overline{\sigma }}} \rightarrow {\mathbb {R}},\, ({\mathbf {x}}, {\mathbf {M}}) \mapsto {{\,\mathrm{det}\,}}\big ({\mathbf {T}}_1({\mathbf {x}}, {\mathbf {M}})\big ) = {{\,\mathrm{det}\,}}{\mathbf {M}}\end{aligned}$$

is infinitely Fréchet differentiable with

$$\begin{aligned} \big \Vert {{\,\mathrm{D}\,}}^r T_5({\mathbf {x}}, {\mathbf {M}})\big \Vert _{{\mathcal {B}}^r({\mathbb {R}}^d \times {\mathbb {R}}^{d \times d}; {\mathbb {R}})} \le r! k_5 c_5^r \end{aligned}$$

for all $({\mathbf {x}}, {\mathbf {M}}) \in {\mathcal {D}}\times {\mathbb {R}}^{d \times d}_{{\underline{\sigma }}, {\overline{\sigma }}}$, and $k_5 = (2 {\overline{\sigma }})^d$, $c_5 = {\overline{\sigma }} + 1$.

Finally, the use of [30, Lemma 3] yields the assertion, as ${\mathbf {T}}({\mathbf {x}}, {\mathbf {M}}) = T_5({\mathbf {x}}, {\mathbf {M}}) {\mathbf {T}}_4({\mathbf {x}}, {\mathbf {M}})$. $\square$

#### Lemma 4

The mapping *s* is infinitely Fréchet differentiable with

$$\begin{aligned} \big \Vert {{\,\mathrm{D}\,}}^r s({\mathbf {x}}, {\mathbf {M}})\big \Vert _{{\mathcal {B}}^r({\mathbb {R}}^d \times {\mathbb {R}}^{d \times d}; {\mathbb {R}})} \le r! k_s c_s^r \end{aligned}$$

for all $({\mathbf {x}}, {\mathbf {M}}) \in {\mathcal {D}}\times {\mathbb {R}}^{d \times d}_{{\underline{\sigma }}, {\overline{\sigma }}}$ with $k_s = (2 {\overline{\sigma }})^d k_f$ and $c_s = 2 \max \big \{c_f \displaystyle \max _{{\mathbf {x}}\in {\mathcal {D}}} \big \Vert {\mathbf {x}}\big \Vert , {\overline{\sigma }}\big \} + 2$, where $k_f$, $c_f$ are constants such that $\big \Vert {{\,\mathrm{D}\,}}^r f({\mathbf {x}})\big \Vert _{{\mathcal {B}}^r({\mathbb {R}}^d; {\mathbb {R}})} \le r! k_f c_f^r$ holds for all ${\mathbf {x}}\in {\mathcal {D}}$.

#### Proof

We start with the mapping

$$\begin{aligned} {\mathbf {s}}_1 :{\mathcal {D}}\times {\mathbb {R}}^{d \times d}_{{\underline{\sigma }}, {\overline{\sigma }}} \rightarrow {\mathcal {D}},\, ({\mathbf {x}}, {\mathbf {M}}) \mapsto {\mathbf {x}}, \end{aligned}$$

which is infinitely Fréchet differentiable with

$$\begin{aligned} \big \Vert {{\,\mathrm{D}\,}}^r {\mathbf {s}}_1({\mathbf {x}}, {\mathbf {M}})\big \Vert _{{\mathcal {B}}^r({\mathbb {R}}^d \times {\mathbb {R}}^{d \times d}; {\mathbb {R}}^d)} \le r! k_1 c_1^r \end{aligned}$$

for all $({\mathbf {x}}, {\mathbf {M}}) \in {\mathcal {D}}\times {\mathbb {R}}^{d \times d}_{{\underline{\sigma }}, {\overline{\sigma }}}$, and $k_1 = \displaystyle \max _{{\mathbf {x}}\in {\mathcal {D}}} \big \Vert {\mathbf {x}}\big \Vert$, $c_1 = 1$. Then, using [30, Lemma 4], we see that the mapping

$$\begin{aligned} s_2 :{\mathcal {D}}\times {\mathbb {R}}^{d \times d}_{{\underline{\sigma }}, {\overline{\sigma }}} \rightarrow {\mathbb {R}},\, ({\mathbf {x}}, {\mathbf {M}}) \mapsto f\big ({\mathbf {s}}_1({\mathbf {x}}, {\mathbf {M}})\big ) = f({\mathbf {x}}) \end{aligned}$$

is infinitely Fréchet differentiable with

$$\begin{aligned} \big \Vert {{\,\mathrm{D}\,}}^r s_2({\mathbf {x}}, {\mathbf {M}})\big \Vert _{{\mathcal {B}}^r({\mathbb {R}}^d \times {\mathbb {R}}^{d \times d}; {\mathbb {R}})} \le r! k_2 c_2^r \end{aligned}$$

for all $({\mathbf {x}}, {\mathbf {M}}) \in {\mathcal {D}}\times {\mathbb {R}}^{d \times d}_{{\underline{\sigma }}, {\overline{\sigma }}}$, and $k_2 = k_f$, $c_2 = c_f \displaystyle \max _{{\mathbf {x}}\in {\mathcal {D}}} \big \Vert {\mathbf {x}}\big \Vert + 1$.

Moreover, as shown in the previous proof we also have that

$$\begin{aligned} s_3 :{\mathcal {D}}\times {\mathbb {R}}^{d \times d}_{{\underline{\sigma }}, {\overline{\sigma }}} \rightarrow {\mathbb {R}},\, ({\mathbf {x}}, {\mathbf {M}}) \mapsto {{\,\mathrm{det}\,}}{\mathbf {M}}\end{aligned}$$

is infinitely Fréchet differentiable with

$$\begin{aligned} \big \Vert {{\,\mathrm{D}\,}}^r s_3({\mathbf {x}}, {\mathbf {M}})\big \Vert _{{\mathcal {B}}^r({\mathbb {R}}^d \times {\mathbb {R}}^{d \times d}; {\mathbb {R}})} \le r! k_3 c_3^r \end{aligned}$$

for all $({\mathbf {x}}, {\mathbf {M}}) \in {\mathcal {D}}\times {\mathbb {R}}^{d \times d}_{{\underline{\sigma }}, {\overline{\sigma }}}$, and $k_3 = (2 {\overline{\sigma }})^d$, $c_3 = {\overline{\sigma }} + 1$. Lastly, the use of [30, Lemma 3] yields the assertion, as $s({\mathbf {x}}, {\mathbf {M}}) = s_2({\mathbf {x}}, {\mathbf {M}}) s_3({\mathbf {x}}, {\mathbf {M}})$. $\square$

Now, these results enable us to show the following regularity estimates for the diffusion coefficient ${\hat{{\mathbf {A}}}}$ and the right hand side ${\hat{f}}$.

#### Theorem 1

We know for all ${\varvec{\alpha }}\in {\mathbb {N}}^{{\mathbb {N}}^*}_f$ that

$$\begin{aligned} \big |\big |\big |\partial _{\mathbf {y}}^{\varvec{\alpha }}{\hat{{\mathbf {A}}}}\big |\big |\big |_{\tau , \infty , D; {\mathbb {R}}_\mathrm {symm}^{d \times d}} \le \big |{\varvec{\alpha }}\big |! k_{{\hat{{\mathbf {A}}}}} c_{{\hat{{\mathbf {A}}}}}^{|{{\varvec{\alpha }}}|} {\varvec{\gamma }}^{{\varvec{\alpha }}} \quad \text {and}\quad \big |\big |\big |\partial _{\mathbf {y}}^{\varvec{\alpha }}{\hat{f}}\big |\big |\big |_{\tau -1, 2, D; {\mathbb {R}}} \le \big |{\varvec{\alpha }}\big |! k_{{\hat{f}}} c_{{\hat{f}}}^{|{{\varvec{\alpha }}}|} {\varvec{\gamma }}^{{\varvec{\alpha }}} , \end{aligned}$$

where

$$\begin{aligned} k_{{\hat{{\mathbf {A}}}}}&\mathrel {\mathrel {\mathop :}=}k_{\mathbf {T}}\sum _{r=0}^{\tau } 2^r c_{\mathbf {T}}^r k_{{\mathbf {V}}{\mathbf {J}}}^r \, ,&c_{{\hat{{\mathbf {A}}}}}&\mathrel {\mathrel {\mathop :}=}2 c_{\mathbf {T}}k_{{\mathbf {V}}{\mathbf {J}}} + 1 \, , \\ k_{{\hat{f}}}&\mathrel {\mathrel {\mathop :}=}\sqrt{\frac{\big |{\mathcal {D}}\big |}{{\underline{\sigma }}^{d}}} k_s \sum _{r=0}^{\tau } 2^r c_s^r k_{{\mathbf {V}}{\mathbf {J}}}^r&\text {and}\quad c_{{\hat{f}}}&\mathrel {\mathrel {\mathop :}=}2 c_s k_{{\mathbf {V}}{\mathbf {J}}} + 1 . \end{aligned}$$

#### Proof

Because ${\hat{{\mathbf {A}}}} = {\mathbf {T}}\circ ({\mathbf {V}}, {\mathbf {J}})$, we can employ [30, Lemma 8] to arrive at

$$\begin{aligned}&\big |\big |\big |{\hat{{\mathbf {A}}}}\big |\big |\big |_{\tau , \infty , D; {\mathbb {R}}_\mathrm {symm}^{d \times d}} \\&\quad \le \sum _{r=0}^{\tau } \frac{1}{r!} \big |\big |\big |{{\,\mathrm{D}\,}}^{r} {\mathbf {T}}\circ ({\mathbf {V}}, {\mathbf {J}})\big |\big |\big |_{\infty , D; {\mathcal {B}}^{r}({\mathbb {R}}^d \times {\mathbb {R}}^{d \times d}; {\mathbb {R}}_\mathrm {symm}^{d \times d}))} \big |\big |\big |({\mathbf {V}}, {\mathbf {J}})\big |\big |\big |_{\eta , \tau , D; {\mathbb {R}}^d \times {\mathbb {R}}^{d \times d}}^r \\&\quad \le k_{\mathbf {T}}\sum _{r=0}^{\tau } c_{\mathbf {T}}^r k_{{\mathbf {V}}{\mathbf {J}}}^r \le k_{{\hat{{\mathbf {A}}}}} \end{aligned}$$

as well as, for ${\varvec{\alpha }}\ne {{\varvec{0}}}$,

$$\begin{aligned}&\big |\big |\big |\partial _{\mathbf {y}}^{\varvec{\alpha }}{\hat{{\mathbf {A}}}}\big |\big |\big |_{\tau , \infty , D; {\mathbb {R}}_\mathrm {symm}^{d \times d}} \\&\quad \le {\varvec{\alpha }}! \sum _{s=1}^{|{{\varvec{\alpha }}}|} \frac{1}{s!} \bigg ( \sum _{r=0}^{\tau } \frac{1}{r!} \big |\big |\big |{{\,\mathrm{D}\,}}^{r+s} {\mathbf {T}}\circ ({\mathbf {V}}, {\mathbf {J}})\big |\big |\big |_{\infty , D; {\mathcal {B}}^{r+s}({\mathbb {R}}^d \times {\mathbb {R}}^{d \times d}; {\mathbb {R}}_\mathrm {symm}^{d \times d})} \\&\qquad \qquad \qquad \qquad \big |\big |\big |({\mathbf {V}}, {\mathbf {J}})\big |\big |\big |_{\tau , \infty , D; {\mathbb {R}}^d \times {\mathbb {R}}^{d \times d}}^r\bigg ) \\&\qquad \qquad \sum _{C({\varvec{\alpha }}, s)} \prod _{j=1}^s \frac{1}{{\varvec{\beta }}_j!} \big |\big |\big |\partial _{\mathbf {y}}^{{\varvec{\beta }}_j} ({\mathbf {V}}, {\mathbf {J}})\big |\big |\big |_{\tau , \infty , D; {\mathbb {R}}^d \times {\mathbb {R}}^{d \times d}} \\&\quad \le {\varvec{\alpha }}! \sum _{s=1}^{|{{\varvec{\alpha }}}|} \frac{1}{s!} \bigg ( \sum _{r=0}^{\tau } \frac{1}{r!} (r+s)! k_{\mathbf {T}}c_{\mathbf {T}}^{r+s} k_{{\mathbf {V}}{\mathbf {J}}}^r\bigg ) \sum _{C({\varvec{\alpha }}, s)} \prod _{j=1}^s \frac{1}{{\varvec{\beta }}_j!} k_{{\mathbf {V}}{\mathbf {J}}} {\varvec{\gamma }}^{{\varvec{\beta }}_j} \\&\quad \le {\varvec{\gamma }}^{{\varvec{\alpha }}} k_{\mathbf {T}}\bigg ( \sum _{r=0}^{\tau } 2^r c_{\mathbf {T}}^r k_{{\mathbf {V}}{\mathbf {J}}}^r\bigg ) \sum _{s=1}^{|{{\varvec{\alpha }}}|} 2^s c_{\mathbf {T}}^s k_{{\mathbf {V}}{\mathbf {J}}}^s {\varvec{\alpha }}! \sum _{C({\varvec{\alpha }}, s)} \prod _{j=1}^s \frac{1}{{\varvec{\beta }}_j!} \\&\quad \le {\varvec{\gamma }}^{{\varvec{\alpha }}} \big |{\varvec{\alpha }}\big |! k_{\mathbf {T}}\bigg ( \sum _{r=0}^{\tau } 2^r c_{\mathbf {T}}^r k_{{\mathbf {V}}{\mathbf {J}}}^r\bigg ) \sum _{s=1}^{|{{\varvec{\alpha }}}|} 2^s c_{\mathbf {T}}^s k_{{\mathbf {V}}{\mathbf {J}}}^s \left( {\begin{array}{c}|{{\varvec{\alpha }}}| - 1\\ s - 1\end{array}}\right) \\&\quad \le {\varvec{\gamma }}^{{\varvec{\alpha }}} \big |{\varvec{\alpha }}\big |! k_{\mathbf {T}}\bigg ( \sum _{r=0}^{\tau } 2^r c_{\mathbf {T}}^r k_{{\mathbf {V}}{\mathbf {J}}}^r\bigg ) (2 c_{\mathbf {T}}k_{{\mathbf {V}}{\mathbf {J}}} + 1)^{|{{\varvec{\alpha }}}|} , \end{aligned}$$

where we make use of the combinatorial identity shown in Lemma 1 yielding the bound (9).

This proves the assertion for ${\hat{{\mathbf {A}}}}$, while the assertion for ${\hat{f}}$ follows analogously after remarking that

$$\begin{aligned}&\big |\big |\big |{{\,\mathrm{D}\,}}^{r} s \circ ({\mathbf {V}}, {\mathbf {J}})\big |\big |\big |_{2, D; {\mathcal {B}}^{r}({\mathbb {R}}^d \times {\mathbb {R}}^{d \times d}; {\mathbb {R}})} \\&\quad = \mathop {\hbox {ess sup}}\limits _{{\mathbf {y}}\in \square } \big \Vert {{\,\mathrm{D}\,}}^r s \circ ({\mathbf {V}}[{\mathbf {y}}], {\mathbf {J}}[{\mathbf {y}}])\big \Vert _{2, D; {\mathcal {B}}^{r}({\mathbb {R}}^d \times {\mathbb {R}}^{d \times d}; {\mathbb {R}})} \\&\quad \le \mathop {\hbox {ess sup}}\limits _{{\mathbf {y}}\in \square } \big \Vert {{\,\mathrm{D}\,}}^r s\big \Vert _{2, {\mathfrak {D}}[{\mathbf {y}}]; {\mathcal {B}}^{r}({\mathbb {R}}^d \times {\mathbb {R}}^{d \times d}; {\mathbb {R}})} \sqrt{{\underline{\sigma }}^{-d}} \\&\quad \le \mathop {\hbox {ess sup}}\limits _{{\mathbf {y}}\in \square } \big \Vert {{\,\mathrm{D}\,}}^r s\big \Vert _{\infty , {\mathfrak {D}}[{\mathbf {y}}]; {\mathcal {B}}^{r}({\mathbb {R}}^d \times {\mathbb {R}}^{d \times d}; {\mathbb {R}})} \sqrt{\big |{\mathfrak {D}}[{\mathbf {y}}]\big | {\underline{\sigma }}^{-d}} \\&\quad \le \big \Vert {{\,\mathrm{D}\,}}^r s\big \Vert _{\infty , {\mathcal {D}}; {\mathcal {B}}^{r}({\mathbb {R}}^d \times {\mathbb {R}}^{d \times d}; {\mathbb {R}})} \sqrt{\big |{\mathcal {D}}\big | {\underline{\sigma }}^{-d}} \\&\quad \le r! \sqrt{\big |{\mathcal {D}}\big |{\underline{\sigma }}^{-d}} k_s c_s^r . \end{aligned}$$

$$\square $$

### Parametric regularity of the solution

It follows from [20, Propositions 3.2.1.2 and 3.1.3.1], when $\tau = 1$ and *D* is convex and bounded, or from [13, Theorem 8.13], when *D* is of class $C^{\tau +1}$, that for almost any ${\mathbf {y}}\in \square$ we have ${\hat{u}}[{\mathbf {y}}] \in H^{\tau +1}(D)$ with

$$\begin{aligned} \big \Vert {\hat{u}}[{\mathbf {y}}]\big \Vert _{\tau +1, 2, D} \le C_{er} \big \Vert {\hat{f}}[{\mathbf {y}}]\big \Vert _{\tau -1, 2, D} , \end{aligned}$$

where $C_{er}$ only depends on *D*, ${\underline{\sigma }}$, ${\overline{\sigma }}$, $\tau$ and $c_{{\varvec{\gamma }}}$. This obviously directly implies the following result.

#### Lemma 5

The unique solution ${\hat{u}} \in L_{{\mathbb {P}}_{\mathbf {y}}}^\infty \big (\square ; H_0^1(D)\big )$ of (6) indeed also fulfils ${\hat{u}} \in L_{{\mathbb {P}}_{\mathbf {y}}}^\infty \big (\square ; H^{\tau +1}(D)\big )$, with

$$\begin{aligned} \big |\big |\big |{\hat{u}}\big |\big |\big |_{\tau +1, 2, D} \le C_{er} \big |\big |\big |{\hat{f}}\big |\big |\big |_{\tau -1, 2, D} . \end{aligned}$$

Moreover, this higher spatial regularity also carries over to the derivates $\partial _{\mathbf {y}}^{\varvec{\alpha }}{\hat{u}}$.^9^

#### Theorem 2

The derivatives of the solution ${\hat{u}}$ of (6) satisfy

$$\begin{aligned} \big |\big |\big |\partial _{\mathbf {y}}^{\varvec{\alpha }}{\hat{u}}\big |\big |\big |_{\tau +1, 2, D}&\le \big |{\varvec{\alpha }}\big |! c^{|{{\varvec{\alpha }}}|+1} {\varvec{\gamma }}^{{\varvec{\alpha }}}, \end{aligned}$$

where $$c \mathrel {\mathrel {\mathop :}=}\max \big \{2, 3 C_{er} \tau ^2 d^2 k_{{\hat{{\mathbf {A}}}}}, 3 C_{er} k_{{\hat{f}}}\big \} \max \big \{c_{{\hat{f}}}, c_{{\hat{{\mathbf {A}}}}}\big \}$$.

## The coupling of FEM and BEM

**Fig. 1:**
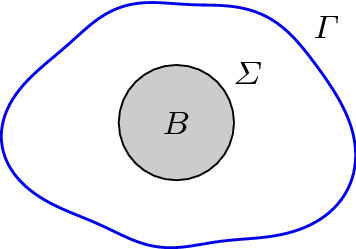
The domain *D*, the subdomain *B*, and the boundaries $\varGamma = \partial D$ and $\varSigma = \partial B$

While the results in the previous subsections are valid for general random domain mappings, we will now restrict them according to the remarks made in the introduction. That is, we assume for the rest of the article that we are given a random boundary description, $\varGamma [{\mathbf {y}}]$, and the fixed, deterministic subdomain *B*, which describe our random domain, compare Fig. 1 when $\varGamma = \varGamma [{\mathbf {y}}]$.

We will assume that there is a random domain mapping ${\mathbf {V}}$ which fulfils the Assumption 1 as well as fulfilling ${\mathbf {V}}[{\mathbf {y}}]|_B = {{\,\mathrm{Id}\,}}_B$ and ${\mathbf {V}}[{\mathbf {y}}](\partial D) = \varGamma [{\mathbf {y}}]$ for almost any ${\mathbf {y}}$. Then, we know from the previous section that ${\hat{u}} :\square \rightarrow H^{\tau +1}(D)$ is analytic which also implies that ${\mathcal {F}}\circ u|_B :\square \rightarrow {\mathcal {X}}$ is analytic.

So, to be able to use multilevel quadrature to compute the quantity of interest efficiently, we consider a formulation here, that enables us to compute the Galerkin solution $u_{h}[{\mathbf {y}}] \in H^1(B)$ with a mesh on *B* but without needing a mesh on ${\mathfrak {D}}[{\mathbf {y}}] \setminus B$ or needing the knowledge of the random domain mapping. Similiar to the approach in [12], one arrives at such a formulation by reformulating the boundary value problem as two coupled problems involving only boundary integral equations on the random boundary $\varGamma [{\mathbf {y}}]$, see for example [8, 23], and then discretising the variational formulation of that formulation with a Galerkin approach, along the lines of [25].

### Newton potential

For sake of simplicity in representation, we shall restrict ourselves in this and the following subsections to the deterministic boundary value problem

13 $$\begin{aligned} -\Delta u = f\ \text {in }D, \quad u = 0\ \text {on } \varGamma \mathrel {\mathrel {\mathop :}=}\partial D, \end{aligned}$$

i.e., the domain *D* is assumed to be fixed. Of course, when applying a sampling method for (1), the underlying domains are always different. In order to resolve the inhomogeneity in (13), we introduce a Newton potential ${\mathcal {N}}_f$ which satisfies

14 $$\begin{aligned} -\Delta {\mathcal {N}}_f = f \quad \text {in }{\widetilde{D}}. \end{aligned}$$

Here, ${\widetilde{D}}$ is a sufficiently large domain containing ${\mathfrak {D}}[{\mathbf {y}}]$ almost surely.

The Newton potential is supposed to be explicitly known like in our numerical example (see Sect. 6) or computed with sufficiently high accuracy. Especially, since the domain ${\widetilde{D}}$ can be chosen fairly simple, one can apply finite elements based on tensor products of higher order spline functions (in $[-R,R]^d$) or dual reciprocity methods. Notice that the Newton potential has to be computed only once in advance.

By making the ansatz

15 $$\begin{aligned} u = {\mathcal {N}}_f + {\tilde{u}} \end{aligned}$$

and setting ${\tilde{g}} \mathrel {\mathrel {\mathop :}=}-{\mathcal {N}}_f$, we arrive at the problem of seeking a harmonic function ${\tilde{u}}$ which solves the following Dirichlet problem for the Laplacian

16 $$\begin{aligned} \Delta {\tilde{u}} = 0\ \text {in }D,\quad {\tilde{u}} = {\tilde{g}}\ \text {on }\varGamma . \end{aligned}$$

Now, we are able to apply the coupling of finite elements and boundary elements.

### Reformulation as a coupled problem

For the subdomain $B \subset D$, we set $\varSigma \mathrel {\mathrel {\mathop :}=}\partial B$, see Fig. 1 for an illustration. The normal vectors ${\mathbf {n}}$ at $\varGamma$ and $\varSigma$ are assumed to point into $D\setminus {\overline{B}}$. We shall split (16) in two coupled boundary value problems in accordance with17

In order to derive suitable boundary integral equations for the problem in $D\setminus {\overline{B}}$, we define the single layer operator ${\mathcal {V}}_{\varPhi \varPsi }$, the double layer operator ${\mathcal {K}}_{\varPhi \varPsi }$ and its adjoint ${\mathcal {K}}_{\varPsi \varPhi }^\star$, and the hypersingular operator ${\mathcal {W}}_{\varPhi \varPsi }$ with respect to the boundaries $\varPhi ,\varPsi \in \{\varGamma ,\varSigma \}$ by

$$\begin{aligned} \left. \begin{aligned} ({\mathcal {V}}_{\varPhi \varPsi }v)({\mathbf {x}})&\mathrel {\mathrel {\mathop :}=}\int _\varPhi G({\mathbf {x}},{\mathbf {z}})v({\mathbf {z}}){{\,\mathrm{d}\,}}\!\sigma _{\mathbf {z}}, \\ ({\mathcal {K}}_{\varPhi \varPsi }v)({\mathbf {x}})&\mathrel {\mathrel {\mathop :}=}\int _\varPhi \frac{\partial G({\mathbf {x}},{\mathbf {z}})}{\partial {\mathbf {n}}({\mathbf {z}})} v({\mathbf {z}}){{\,\mathrm{d}\,}}\!\sigma _{\mathbf {z}}, \\ ({\mathcal {K}}_{\varPhi \varPsi }^\star v)({\mathbf {x}})&\mathrel {\mathrel {\mathop :}=}\int _\varPhi \frac{\partial }{\partial {\mathbf {n}}({\mathbf {x}})} G({\mathbf {x}},{\mathbf {z}})v({\mathbf {z}}){{\,\mathrm{d}\,}}\!\sigma _{\mathbf {z}}, \\ ({\mathcal {W}}_{\varPhi \varPsi }v)({\mathbf {x}})&\mathrel {\mathrel {\mathop :}=}- \frac{\partial }{\partial {\mathbf {n}}({\mathbf {x}})} \int _\varPhi \frac{\partial G({\mathbf {x}},{\mathbf {z}})}{\partial {\mathbf {n}}({\mathbf {z}})}v({\mathbf {z}}){{\,\mathrm{d}\,}}\!\sigma _{\mathbf {z}}, \end{aligned} \quad \right\} \quad {\mathbf {x}}\in \varPsi . \end{aligned}$$

Here, $G({\mathbf {x}},{\mathbf {z}})$ denotes the fundamental solution of the Laplacian which is given by

$$\begin{aligned} G({\mathbf {x}},{\mathbf {z}}) = {\left\{ \begin{array}{ll} -\frac{1}{2\pi } \log \big \Vert {\mathbf {x}}-{\mathbf {z}}\big \Vert , &{} d=2 , \\ \frac{1}{4\pi \log \Vert {{\mathbf {x}}-{\mathbf {z}}}\Vert }, &{} d=3 . \end{array}\right. } \end{aligned}$$

By introducing the variables $\sigma _\varSigma \mathrel {\mathrel {\mathop :}=}({\partial {\tilde{u}}} /{\partial {\mathbf {n}}})|_\varSigma$ and $\sigma _\varGamma \mathrel {\mathrel {\mathop :}=}({\partial {\tilde{u}}} /{\partial {\mathbf {n}}})|_\varGamma$, the coupled system (17) yields the following nonlocal boundary value problem: Find $({\tilde{u}},\sigma _\varSigma ,\sigma _\varGamma )$ such that

$$\begin{aligned} \begin{aligned} \Delta {\tilde{u}}&= 0&\qquad&\text {in } B , \\ {\mathcal {W}}_{\varSigma \varSigma }{\tilde{u}} + \sigma _\varSigma + \Big ({\mathcal {K}}_{\varSigma \varSigma }^\star - \frac{1}{2}\Big )\sigma _\varSigma + {\mathcal {K}}_{\varGamma \varSigma }^\star \sigma _\varGamma&= - {\mathcal {W}}_{\varGamma \varSigma }{\tilde{g}}&\qquad&\text {on } \varSigma , \\ \Big (\frac{1}{2}-{\mathcal {K}}_{\varSigma \varSigma }\Big ){\tilde{u}} + {\mathcal {V}}_{\varSigma \varSigma }\sigma _\varSigma + {\mathcal {V}}_{\varGamma \varSigma }\sigma _\varGamma&= {\mathcal {K}}_{\varGamma \varSigma }{\tilde{g}}&\qquad&\text {on } \varSigma , \\ - {\mathcal {K}}_{\varSigma \varGamma }{\tilde{u}} + {\mathcal {V}}_{\varSigma \varGamma }\sigma _\varSigma + {\mathcal {V}}_{\varGamma \varGamma }\sigma _\varGamma&= \Big ({\mathcal {K}}_{\varGamma \varGamma } - \frac{1}{2}\Big ){\tilde{g}}&\qquad&\text {on } \varGamma . \end{aligned} \end{aligned}$$

This system is the so-called *two integral formulation*, which is equivalent to our original model problem (16), see for example [8, 23].

### Variational formulation

We next introduce the product space ${\mathcal {H}}\mathrel {\mathrel {\mathop :}=}H^1(B)\times H^{-1/2}(\varSigma )\times H^{-1/2}(\varGamma )$, equipped by the product norm

$$\begin{aligned} \big \Vert (v,\sigma _\varSigma ,\sigma _\varGamma )\big \Vert _{{\mathcal {H}}}^2 \mathrel {\mathrel {\mathop :}=}\big \Vert v\big \Vert _{H^1(B)}^2 + \big \Vert \sigma _\varSigma \big \Vert _{H^{-1/2}(\varSigma )}^2 + \big \Vert \sigma _\varGamma \big \Vert _{H^{-1/2}(\varGamma )}^2 . \end{aligned}$$

Further, let $a :{\mathcal {H}}\times {\mathcal {H}}\rightarrow {\mathbb {R}}$, be the bilinear form defined by

$$\begin{aligned} \begin{aligned}&a\bigg ((v,\sigma _\varSigma ,\sigma _\varGamma ),(w,\lambda _\varSigma ,\lambda _\varGamma )\bigg ) = (\nabla w, \nabla v)_{L^2(B)} \\&\qquad + \left( \left[ \begin{array}{l}w \\ \lambda _\varSigma \\ \lambda _\varGamma \end{array}\right] , \left[ \begin{array}{l@{\quad }l@{\quad }l} {\mathcal {W}}_{\varSigma \varSigma } &{} {\mathcal {K}}_{\varSigma \varSigma }^\star - 1/2 &{} {\mathcal {K}}_{\varGamma \varSigma }^\star \\ 1/2-{\mathcal {K}}_{\varSigma \varSigma } &{} {\mathcal {V}}_{\varSigma \varSigma } &{} {\mathcal {V}}_{\varGamma \varSigma } \\ -{\mathcal {K}}_{\varSigma \varGamma } &{} {\mathcal {V}}_{\varSigma \varGamma } &{} {\mathcal {V}}_{\varGamma \varGamma } \end{array}\right] \left[ \begin{array}{l} v \\ \sigma _\varSigma \\ \sigma _\varGamma \end{array}\right] \right) _{{\mathcal {L}}} , \end{aligned} \end{aligned}$$

where ${\mathcal {L}}\mathrel {\mathrel {\mathop :}=}L^2(\varSigma ) \times L^2(\varSigma ) \times L^2(\varGamma )$. For sake of simplicity in representation, we omitted the trace operator in expressions like $(w,{\mathcal {W}}_{\varSigma \varSigma }v)_{L^2(\varSigma )}$ etc.

Introducing the linear functional $F :{\mathcal {H}}\rightarrow {\mathbb {R}}$,

$$\begin{aligned} F(w,\lambda _\varSigma ,\lambda _\varGamma ) =\left( \left[ \begin{array}{l} w \\ \lambda _\varSigma \\ \lambda _\varGamma \end{array}\right] , \left[ \begin{array}{c} -{\mathcal {W}}_{\varGamma \varSigma } \\ {\mathcal {K}}_{\varGamma \varSigma } \\ {\mathcal {K}}_{\varGamma \varGamma }-1/2 \end{array}\right] {\tilde{g}}\right) _{{\mathcal {L}}} , \end{aligned}$$

the variational formulation is given by: Seek $({\tilde{u}},\sigma _\varSigma ,\sigma _\varGamma )\in {\mathcal {H}}$ such that

18 $$\begin{aligned} a\big (({\tilde{u}},\sigma _\varSigma ,\sigma _\varGamma ),(w,\lambda _\varSigma ,\lambda _\varGamma )\big ) = F(w,\lambda _\varSigma ,\lambda _\varGamma ) \end{aligned}$$

for all $(w,\lambda _\varSigma ,\lambda _\varGamma )\in {\mathcal {H}}$. In accordance with [12, Theorem 4.1], the variational formulation (18) admits a unique solution $({\tilde{u}},\sigma _\varSigma ,\sigma _\varGamma )\in {\mathcal {H}}$ for all $F\in {\mathcal {H}}'$, provided that *D* has a conformal radius which is smaller than one if $d=2$.

### Galerkin discretisation

Since the variational formulation is stable without further restrictions, the discretisation is along the lines of [25]. We first introduce a uniform triangulation of *B* which in turn induces a uniform triangulation of $\varSigma$. Moreover, we introduce a uniform triangulation of the boundary $\varGamma$. Note, that the precise approach used to mesh $\varGamma$ in applications will depend on which description of the random boundary is given. However, as some form of description of the random boundary must be available, it generally will be easier to mesh it, as opposed to meshing the whole domain, cf. for example [24]. Indeed, if the random boundary is given as a star-shaped parametrisation or if it is given by a random boundary mapping, a mesh on the *d*-sphere or reference boundary may be used to construct triangulations on all sampled boundaries. On the other hand, if the random boundary is described by some (parametric or geometric) surface mesh, coming for example from some computer assisted design system, which is perturbed by moving control points or mesh vertices, then this immeadiately supplies triangulations on all sampled boundaries.

We define the maximum diameter of all elements of the triangulation of *B* and of the surface triangulation of $\varGamma$ by *h*. For the FEM part, we consider continuous, piecewise linear ansatz functions $\{\varphi _1^B, \ldots , \varphi _{n_\text {dof}^B}^B\}$ with respect to the given domain mesh. For the BEM part, we employ piecewise constant ansatz functions $\{\psi _1^\varPhi , \ldots , \psi _{m_\text {dof}^\varPhi }^\varPhi \}$ on the respective triangulations of the boundaries $\varPhi \in \{\varSigma ,\varGamma \}$.

For sake of simplicity in representation, we set $\varphi _k^\varSigma \mathrel {\mathrel {\mathop :}=}\varphi _k^B|_\varSigma$ for all $k = 1, \ldots , n_\text {dof}^B$. Note that most of these functions vanish except for those with nonzero trace which coincide with continuous, piecewise linear ansatz functions on $\varSigma$. Finally, we shall introduce the set of continuous, piecewise linear ansatz functions on the triangulation of $\varGamma$, which we denote by $\{\varphi _1^\varGamma , \ldots , \varphi _{m_\text {dof}^\varGamma }^\varGamma \}$, where we have $m_\text {dof}^\varGamma \sim n_\text {dof}^\varGamma$.

Then, introducing the system matrices

$$\begin{aligned} \begin{aligned} {\mathbf {A}}&= \begin{bmatrix} (\nabla \varphi _{k'}^B, \nabla \varphi _{k}^B)_{L^2(B)} \end{bmatrix}_{k,k'} ,&\qquad {\mathbf {W}}_{\varPhi \varPsi }&= \begin{bmatrix} ({\mathcal {W}}_{\varPhi \varPsi }\varphi _{k'}^\varPhi , \varphi _{k}^\varPsi )_{L^2(\varPsi )} \end{bmatrix}_{k,k'} , \\ {\mathbf {B}}_{\varPhi }&= \begin{bmatrix} \frac{1}{2} (\varphi _{k'}^\varPhi , \psi _{k}^\varPhi )_{L^2(\varPhi )} \end{bmatrix}_{k,k'} ,&\qquad {\mathbf {K}}_{\varPhi \varPsi }&= \begin{bmatrix} ({\mathcal {K}}_{\varPhi \varPsi }\varphi _{k'}^\varPhi , \psi _{k}^\varPsi )_{L^2(\varPsi )} \end{bmatrix}_{k,k'} , \\ {\mathbf {G}}_{\varPhi }&= \begin{bmatrix} (\varphi _{k'}^\varPhi ,\varphi _{k}^\varPhi )_{L^2(\varPhi )} \end{bmatrix}_{k,k'} ,&\qquad {\mathbf {V}}_{\varPhi \varPsi }&= \begin{bmatrix} ({\mathcal {V}}_{\varPhi \varPsi }\psi _{k'}^{\varPhi }, \psi _{k}^{\varPsi })_{L^2(\varPsi )} \end{bmatrix}_{k,k'} , \end{aligned} \end{aligned}$$

where again $\varPhi , \varPsi \in \{\varSigma ,\varGamma \}$, and the data vector

$$\begin{aligned} {\mathbf {g}}= \begin{bmatrix} ({\tilde{g}}, \varphi _{k}^\varGamma )_{L^2(\varGamma )} \end{bmatrix}_{k} , \end{aligned}$$

we obtain the following linear system of equations

19 $$\begin{aligned} \begin{bmatrix} {\mathbf {A}}+ {\mathbf {W}}_{\varSigma \varSigma } &{} {\mathbf {K}}_{\varSigma \varSigma }^{\mathsf {T}}- {\mathbf {B}}_\varSigma ^{\mathsf {T}}&{} {\mathbf {K}}_{\varSigma \varGamma }^{\mathsf {T}}\\ {\mathbf {B}}_\varSigma - {\mathbf {K}}_{\varSigma \varSigma } &{} {\mathbf {V}}_{\varSigma \varSigma } &{} {\mathbf {V}}_{\varGamma \varSigma } \\ - {\mathbf {K}}_{\varSigma \varGamma } &{} {\mathbf {V}}_{\varSigma \varGamma } &{} {\mathbf {V}}_{\varGamma \varGamma } \end{bmatrix} \begin{bmatrix} {\mathbf {u}}\\ {\varvec{\sigma }}_\varSigma \\ {\varvec{\sigma }}_\varGamma \end{bmatrix} = \begin{bmatrix} -{\mathbf {W}}_{\varGamma \varSigma } \\ {\mathbf {K}}_{\varGamma \varSigma } \\ {\mathbf {K}}_{\varGamma \varGamma }-{\mathbf {B}}_\varGamma \end{bmatrix} {\mathbf {G}}_\varGamma ^{-1} {\mathbf {g}}. \end{aligned}$$

We mention that ${\mathbf {G}}_\varGamma ^{-1}{\mathbf {g}}$ corresponds to the $L^2(\varGamma )$-orthogonal projection of the given Dirichlet data ${\tilde{g}} \in H^{1/2}(\varGamma )$ onto the space of the continuous, piecewise linear ansatz functions on $\varGamma$. That way, we can also apply fast boundary element techniques to the boundary integral operators on the right hand side of the system (19) of linear equations.

By applying standard error estimates for the Galerkin scheme and possibly also the Aubin–Nitsche trick, see for example [12, Proposition 4.1], the present discretisation now yields the following error estimate.^10^

#### Proposition 2

We denote the solution of (18) by $({{\tilde{u}}},\sigma _\varSigma , \sigma _\varGamma )$ and the Galerkin solution by $({{\tilde{u}}}_h, \sigma _{\varSigma ,h},\sigma _{\varGamma ,h})$, respectively. Then, we have the error estimates

$$\begin{aligned}&\big \Vert ({\tilde{u}},\sigma _\varSigma ,\sigma _\varGamma ) -({\tilde{u}}_h,\sigma _{\varSigma ,h},\sigma _{\varGamma ,h}) \big \Vert _{H^1(B)\times H^{-1/2}(\varSigma )\times H^{-1/2}(\varGamma )} \\&\quad \lesssim h \big \Vert ({\tilde{u}}, \sigma _\varSigma ,\sigma _\varGamma )\big \Vert _{H^{2}(B)\times H^{1/2}(\varSigma )\times H^{1/2}(\varGamma )} \end{aligned}$$

and

$$\begin{aligned}&\big \Vert ({\tilde{u}},\sigma _\varSigma ,\sigma _\varGamma ) -({\tilde{u}}_h,\sigma _{\varSigma ,h},\sigma _{\varGamma ,h}) \big \Vert _{L^2(B)\times H^{-3/2}(\varSigma )\times H^{-3/2}(\varGamma )} \\&\quad \lesssim h^{2} \big \Vert ({\tilde{u}}, \sigma _\varSigma ,\sigma _\varGamma )\big \Vert _{H^{2}(B)\times H^{1/2}(\varSigma )\times H^{1/2}(\varGamma )} \end{aligned}$$

uniformly in *h*.

### Multigrid based solver for the coupling formulation

To arrive at an efficient solver for the linear system (19) of equations some issues need to be addressed. As we will require a hierarchy of discretisations for the use of the multilevel quadrature method, we introduce a hierarchy of uniform triangulations of *B* and of uniform triangulations of the boundary $\varGamma$ yielded by uniformly refining a given coarse triangulation of *B* and a given coarse triangulation of the boundary $\varGamma$ and enumerated by the level of refinement $\ell \in {\mathbb {N}}$. With this at hand, we consider how to solve the linear system (19) of equations for the $\ell$-th triangulations of *B* and $\varGamma$ in that hierarchy of triangulations.

The complexity is governed by the BEM part since the boundary element matrices are densely populated. Following [25, 26], we apply wavelet matrix compression to reduce this complexity such that the over-all complexity is governed by the FEM part. On the other hand, according to [26, 32], the Bramble–Pasciak–CG (see [2]) provides an efficient and robust iterative solver for the above saddle point system. Combining a nested iteration with the BPX preconditioner (see [3]) for the FEM part and a wavelet preconditioning (see [9, 41]) for the BEM part, we derive an asymptotical optimal solver for the above system, see [26] for the details. We refer the reader to [26] for the details of the implementation of a similar coupling formulation.

## Multilevel quadrature method

The crucial idea of the multilevel quadrature to compute the quantity of interest (5) is to combine an appropriate sequence of quadrature rules for the stochastic variable with a sequence of multilevel discretisations in the spatial variable, for a detailed treaty we refer to [27].

For the spatial approximation, we shall use the hierarchy of triangulations introduced in Subsect. 4.5 to compute the Galerkin solution $u_\ell \in H^1(B)$ on the level $\ell$ triangulations as described there. The Galerkin solution on these triangulations, which by uniform refining have a mesh size $h_\ell \simeq 2^{-\ell }$, thus yield the approximate decomposition

$$\begin{aligned} {\mathcal {F}}(u|_B) \approx {\mathcal {F}}(u_1) + \sum _{\ell =1}^{L-1} \big ({\mathcal {F}}(u_{\ell +1}) - {\mathcal {F}}(u_\ell )\big ) . \end{aligned}$$

Next, we consider a general sequence of quadrature formulas ${\mathbf {Q}}_\ell$ of the form

$$\begin{aligned} \int _{\square } v[{\mathbf {y}}]{{\,\mathrm{d}\,}}\!{\mathbb {P}}_{\mathbf {y}}\approx {\mathbf {Q}}_\ell v = \sum _{i=1}^{N_\ell } \rho _{\ell ,i} v[{\varvec{\xi }}_{\ell ,i}] \end{aligned}$$

with nodes ${\varvec{\xi }}_{\ell ,i}$ and weights $\rho _{\ell ,i}$ for the approximation of the integration over the stochastic variable in its parametrised form ${\mathbf {y}}$. We will assume that the number of points $N_\ell$ of the quadrature formula ${\mathbf {Q}}_\ell$ is chosen such that the corresponding accuracy^11^ is

20 $$\begin{aligned} \varepsilon _\ell \simeq 2^{-\ell }, \quad \ell = 1, \ldots , L . \end{aligned}$$

Consequently, since we can state the quantity of interest as

$$\begin{aligned} {{\,\mathrm{QoI}\,}}(u) = \int _\square {\mathcal {F}}\big (u[{\mathbf {y}}]|_B\big ) {{\,\mathrm{d}\,}}\!{\mathbb {P}}_{\mathbf {y}}\end{aligned}$$

based on the expansion (8), we may approximate it by the multilevel quadrature

21 $$\begin{aligned} {{\,\mathrm{QoI}\,}}^{\text {ml}}_L \mathrel {\mathrel {\mathop :}=}{\mathbf {Q}}_L\big ({\mathcal {F}}(u_1)\big ) + \sum _{\ell =1}^{L-1} {\mathbf {Q}}_{L-\ell }\big ({\mathcal {F}}(u_{\ell +1}) - {\mathcal {F}}(u_\ell )\big ) \end{aligned}$$

as opposed to considering the single-level quadrature

22 $$\begin{aligned} {{\,\mathrm{QoI}\,}}^{\text {sl}}_L \mathrel {\mathrel {\mathop :}=}{\mathbf {Q}}_L\big ({\mathcal {F}}(u_L)\big ) . \end{aligned}$$

Since the multilevel quadrature can be interpreted as a sparse grid approximation, cf. [27], it is known that mixed regularity results of the integrand have to be provided as derived in Section 3, compare [10, 18, 27, 37] for example. Since the mapping $u :\square \rightarrow H^{\tau +1}(B)$ is analytic, we can especially apply the quasi-Monte Carlo method, the Gaussian quadrature, or the sparse grid quadrature, see e.g. [18, 21, 36, 43]. Especially, in case of $H^2$-regularity ($\tau = 1$) and ${\mathcal {F}}= {{\,\mathrm{Id}\,}}_{H^1(B)}$, i.e., ${{\,\mathrm{QoI}\,}}(u) = {\mathbb {E}}(u|_B)$, we then obtain the error estimate, see [27],

23 $$\begin{aligned} \big \Vert {\mathbb {E}}(u|_B) - {{\,\mathrm{QoI}\,}}^{\text {ml}}_L\big \Vert _{H^1(B)} = {\mathcal {O}}(L 2^{-L}) . \end{aligned}$$

As the spatial discretisations employ the hierarchy of triangulations introduced in Subsect. 4.5, which are yielded by uniform refining, the number of degrees of freedom in the linear system (19) for the level $\ell$ triangulations are $\varTheta \big ((2^\ell )^d\big )$. Thus, the linear complexity solver also has $\varTheta \big ((2^\ell )^d\big )$ complexity for one level $\ell$ system to solve, compare [26]. The quadrature formula ${\mathbf {Q}}_\ell$ obviously has a complexity of $\varTheta (N_\ell )$. Now, in view of Theorem 2, we can consider some examples of quadrature methods and explicitly state how $N_\ell$ may be choosen to satisfy the accuracy required in (20).

- If, for example, we assume that there is an $\varepsilon > 0$ such that $\gamma _k \lesssim k^{-3-\varepsilon }$ holds and we consider the quasi-Monte Carlo quadrature based on the Halton sequence, then, we use [28, Lemma 7], a consequence of [43], to see that we may choose
  24 $$\begin{aligned} N_\ell \simeq (2^\ell )^{\frac{1}{1-\delta }} \end{aligned}$$
  for any $$\delta > 0$$.
- Similarily, if we assume that there is a $r > 1$ such that $\gamma _k \lesssim k^{-r}$ holds and we consider the anisotropic sparse grid Gauss–Legendre quadrature, then, we use [21, Theorem 5.7] to see that we may choose
  $$\begin{aligned} N_\ell \simeq (2^\ell )^{\frac{2}{s-1}} \end{aligned}$$
  for any $$s < r$$.

As these quadrature method examples use $N_\ell \simeq (2^\ell )^r$ for some $r > 0$, we will assume this algebraic computational complexity from here on. Thus, the standard single-level quadrature method (22) shows a computational complexity of

$$\begin{aligned} \varTheta \big ((2^L)^r\big ) \varTheta \big ((2^L)^d\big ) = \varTheta \big ((2^L)^{r+d}\big ) , \end{aligned}$$

while the computational complexity of the multilevel quadrature (21) as a sparse grid combination is given by

$$\begin{aligned} \sum _{\ell =0}^{L-1} \varTheta \big ((2^{L-\ell })^r\big ) \varTheta \big ((2^{\ell +1})^d\big ) = {\left\{ \begin{array}{ll} \varTheta \big ((2^L)^{\max (r, d)}\big ) , &{} \text {if } r \ne d , \\ \varTheta \big (L (2^L)^d\big ) , &{} \text {when } r = d , \end{array}\right. } \end{aligned}$$

see e.g. [17]. That is, the computational complexity of the multilevel quadrature (21) is considerably reduced compared to the standard single-level quadrature method (22), which has the same accuracy, see also [1, 7, 27] for example. This is also visible in the numerical example shown in Fig. 3.

### Remark 3

By choosing the accuracy of the quadrature in accordance with $\varepsilon _\ell \simeq 4^{-\ell }$ for $\ell =1,\ldots ,L$ instead of (20), the application of the Aubin–Nitsche trick in Proposition 2 implies the $L^2$-error estimate

25 $$\begin{aligned} \big \Vert {\mathbb {E}}(u|_B) - {{\,\mathrm{QoI}\,}}^{\text {ml}}_L\big \Vert _{L^2(B)} = {\mathcal {O}}(L 4^{-L}) , \end{aligned}$$

when using the same hierarchy of uniform refined triangulations with mesh size $h_\ell \simeq 2^{-\ell }$. To achieve this increased accuracy, (24) must be replaced by

26 $$\begin{aligned} N_\ell = (4^\ell )^{\frac{1}{1-\delta }} \end{aligned}$$

for any $\delta > 0$, and, where applicable, subsequent equations also be modified accordingly. Lastly, we note that the computational complexity of the deterministic solver is not affected when accounting for the $L^2$-error instead of the $H^1$-error.

## Numerical results

**Fig. 2:**
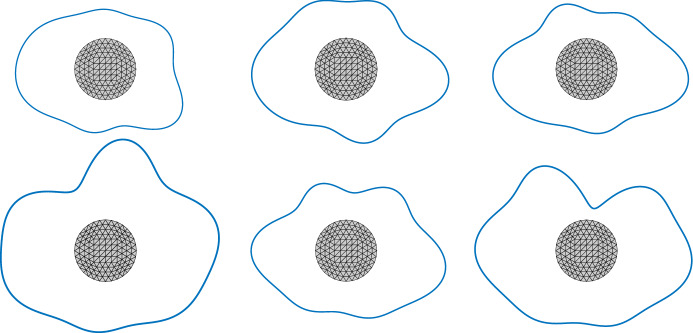
Six samples of the random domain with finite element triangulation of *B* on refinement level 2

In our numerical example, we consider the reference domain *D* to be the ellipse with semi-axis 0.7 and 0.5. We represent its boundary by $\gamma _{\mathrm {ref}} :[0,2\pi ) \rightarrow \partial D$ in polar coordinates and perturb this parametrisation in accordance with

$$\begin{aligned} \gamma [{\mathbf {y}}](\varphi ) = \gamma _{\mathrm {ref}}(\varphi ) + \varepsilon \sum _{j=0}^\infty w_j \big ( y_{-j}\sin (j\varphi ) + y_j\cos (j\varphi )\big ) \end{aligned}$$

where $y_j\in [-0.5,0.5]$ for all $j\in {\mathbb {Z}}$ are independent and identically uniformly distributed random variables and $\varepsilon = 0.05$. The weights $w_j$ are chosen as $w_j = 1$ for all $\big |j\big | \le 5$ and $w_j = (j-5)^{-5.001}$ for all $\big |j\big | > 5$. Hence, we have the decay $\gamma _j \sim j^{-3.001}$ for the choice $\tau = 1$, which is sufficient for applying the quasi-Monte Carlo method based on the Halton sequence, see Sect. 5 and the references [28, 43]. In practice, we set all $w_j$ to zero if $|j|>64$ which corresponds to a dimension truncation after 129 dimensions. The random parametrisation $\gamma [{\mathbf {y}}]$ induces the random domain ${\mathfrak {D}}[{\mathbf {y}}]$. The fixed subset $B \subset D$ is given as the ball of radius 0.2, centered in the origin. For an illustration of six draws, see Fig. 2. We choose $f({\mathbf {x}}) = 1$, for which a suitable Newton potential is then analytically given by ${\mathcal {N}}_f = -(x_1^2+x_2^2)/4$, and consider the $L^2$-tracking type functional

$$\begin{aligned} {{\,\mathrm{QoI}\,}}(u) = {\mathbb {E}}\bigg [\frac{1}{2} \int _B \big |u[{\mathbf {y}}]-{\overline{u}}\big |^2 {{\,\mathrm{d}\,}}\!{\mathbf {x}}\bigg ] \quad \text {with}\quad {\overline{u}}({\mathbf {x}}) = 2 - \bigg (\frac{x_1}{0.4}\bigg )^2 - \bigg (\frac{x_2}{0.3}\bigg )^2 \end{aligned}$$

as quantity of interest.

The coarse triangulation of *B*, based on Zlámal’s curved finite elements [45], consists of 14 curved triangles on the coarse grid, which are then uniformly refined to get the triangulation on the finer grids. The 14 triangles correspond to eight piecewise linear and constant boundary elements each on the boundary $\partial B$. At the boundary $\partial D$, we likewise consider eight piecewise linear and constant boundary elements each on level 0. We then apply successive uniform refinement on the triangulation of *B* and the boundary elements yielding the discretisations of level 1 to 10, with mesh size $h_\ell \simeq 2^{-\ell }$. In order to compute the quantity of interest, we will employ the quasi-Monte Carlo method based on the Halton sequence, see [22] for example, as the quadrature method. For this, essentially^12^ following (24) and (26), we set

27 $$\begin{aligned} N_\ell = 2^{\ell -1} N_1 \end{aligned}$$

and

28 $$\begin{aligned} N_\ell = 4^{\ell -1} N_1, \end{aligned}$$

respectively, with $N_1 = 10, 20, 40$. Thus, $N_1$ is the number of samples the multilevel quadrature uses on the fine grid *L*. Since the exact solution is unknown, we use the quantity of interest computed on level $L = 10$ with $N_\ell = 4^{\ell -1} N_1$ and $N_1 = 40$ as a reference solution.

**Fig. 3:**
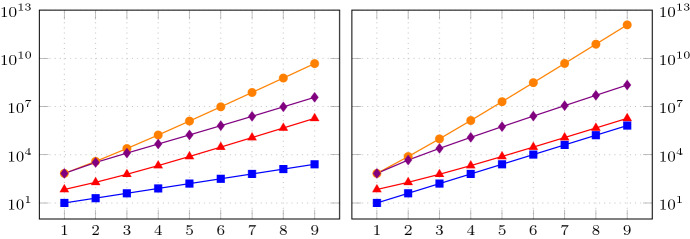
Cost of methods in total number of degrees of freedom (vertical axis) versus maximum level *L* (horizontal axis), when using the number of quadrature points (27) (left) and (28) (right) with $N_1 = 10$. 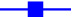 shows the cost of the quadrature, 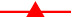 the cost of the FEM-BEM discretisation, 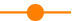 the resulting cost of the single-level and 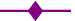 of the multilevel methods

**Fig. 4:**
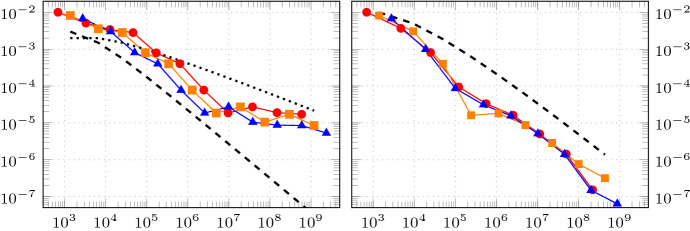
Absolute error of the output functional (vertical axis) versus cost in degrees of freedom (horizontal axis), when using the number of quadrature points (27) (left) and (28) (right). 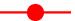 shows the situation with $N_1 = 10$, 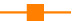 with $$N_1 = 20$$ and 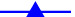 with $$N_1 = 40$$. 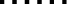 and 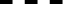 show the asymptotic rates $L 2^{-L}$ and $L 4^{-L}$

The computational costs of these choices are shown in Fig. 3, where the cost is quantified in terms of the total number of degrees of freedom. The FEM-BEM spatial discretisation shows a cost of $\varTheta (4^L)$, while the quadrature discretisation obviously shows costs of $\varTheta (2^L)$ and $\varTheta (4^L)$, respectively. In both settings the multilevel combination, given by (21), seems to show up as having a cost of $\varTheta (4^L)$; however when using the number of quadrature points (28) there is an additional logarithmic factor in the cost, i.e. the cost is $\varTheta (L 4^L)$. For comparison purposes the cost of the single-level approach, as given by (22), is also shown, demonstrating the expected costs of $\varTheta (6^L)$ and $\varTheta (8^L)$, respectively.

As it is seen in Fig. 4, we observe the essentially quadratic convergence rate, when using the number of quadrature points (27). This is in accordance with (25). The situation, when using the number of quadrature points (27), is less clear. The convergence rate is first seemingly quadratic and only then flattens out to be essentially linear, which is what is in accordance with (23). This faster convergence in the preasymptotic regime may be caused by having a spatial discretisation error, which is significantly larger on the coarse triangulations than the error of the coarse quadratures of the quadrature discretisation.

## Conclusion

We provided regularity estimates of the solution to elliptic problems on random domains which allow for the application of many multilevel quadrature methods. In order to avoid the need to compute either a random domain mapping or to generate meshes for every domain sample, we couple finite elements with boundary elements. It has been shown by numerical experiments that this approach is indeed able to exploit the additional regularity we have in the underlying problem without causing numerical problems on too coarse grids.

## Data Availability

The results presented in this article can be replicated solely using the information contained in this article and its references.

## Footnotes

1 We use the function composition ‘$\circ$’ as usual. Moreover, we will only use it for composition in the spatial variable. For example, ${\hat{u}}[\omega ] \mathrel {\mathrel {\mathop :}=}u[\omega ] \circ {\mathbf {V}}[\omega ]$, expands to ${\hat{u}}[\omega ]({\mathbf {x}}) = u[\omega ]\big ({\mathbf {V}}[\omega ]({\mathbf {x}})\big )$.

2 The upper bound on $\sigma$ accounts for the energy space of ${\hat{u}}$.

3 We denote the usual restriction operator, continuously extended by density arguments to the Sobolev spaces, by $\cdot |_B :f \mapsto f|_B$.

4 Care should be taken to not confuse the $\omega$ in $C^{\omega }$, which should be considered pure notation, with a sample of the probability space $\omega \in \varOmega$.

5 The results of this section themselves howeever do not require that the domain mapping fulfils ${\mathbf {V}}[{\mathbf {y}}]\vert _B = {{\,\mathrm{Id}\,}}_B$.

6 It is obviously possible to define ${\mathbf {T}}$ such that it does not depend on ${\mathbf {x}}$. Nevertheless, we include it here so that we can compose both ${\mathbf {T}}$ and *s* directly with $({\mathbf {V}}, {\mathbf {J}})$. Moreover, in general, such when working with an analytic determinstic diffusion coeffcient, cf. Remark 1, it may be necessary to include it.

7 The bound we give here could be reduced to 0 when $\big |{\varvec{\alpha }}\big | > 1$, which possibly can be used to derive smaller bounds in some of the subsequent results, see Remark 2. However, we choose to use it as is, as any tightening of the bound makes it loose this structure, which is also found for more general models of ${\mathbf {V}}$, cf. Remark 1.

8 The formula follows directly from the fact that the determinant function is a polynomial over the entries of the matrix given by the Leibniz formula. Especially, the formula yields an empty sum (of value 0) for $r > d$.

9 We omit the proof, as it is essentially identical to the proof of [30, Theorem 3], apart from the fact that one has to also account for the depenence of ${\hat{f}}$ on ${\mathbf {y}}$, which poses no problems.

10 While the orders chosen for the elements are well-suited when $\tau =1$, higher order elements should be chosen for $\tau > 1$ to reach higher algebraic convergence rates for this error estimate.

11 This choice of accuracy rate is based on the $H^1(B)$-error estimate from the FEM-BEM discretisation, with $h_\ell \simeq 2^{-\ell }$.

12 We ignore the fact that $\delta$ should fulfil $\delta > 0$ and just use $\delta = 0$.
